# Optimal timing of steroid initiation in response to CTLA-4 antibody in metastatic cancer: A mathematical model

**DOI:** 10.1371/journal.pone.0277248

**Published:** 2022-11-10

**Authors:** Nourridine Siewe, Avner Friedman

**Affiliations:** 1 School of Mathematical Sciences, College of Science, Rochester Institute of Technology, Rochester, New York, United States of America; 2 Department of Mathematics, The Ohio State University, Columbus, Ohio, United States of America; RWTH Aachen University Medical Faculty: Rheinisch-Westfalische Technische Hochschule Aachen Medizinische Fakultat, GERMANY

## Abstract

Immune checkpoint inhibitors, introduced in recent years, have revolutionized the treatment of many cancers. However, the toxicity associated with this therapy may cause severe adverse events. In the case of advanced lung cancer or metastatic melanoma, a significant number (10%) of patients treated with CTLA-4 inhibitor incur damage to the pituitary gland. In order to reduce the risk of hypophysitis and other severe adverse events, steroids may be combined with CTLA-4 inhibitor; they reduce toxicity, but they also diminish the anti-cancer effect of the immunotherapy. This trade-off between tumor reduction and the risk of severe adverse events poses the following question: What is the optimal time to initiate treatment with steroid. We address this question with a mathematical model from which we can also evaluate the comparative benefits of each schedule of steroid administration. In particular, we conclude that treatment with steroid should not begin too early, but also not very late, after immunotherapy began; more precisely, it should start as soon as tumor volume, under the effect of CTLA-4 inhibitor alone, begins to decrease. We can also compare the benefits of short term treatment of steroid at high doses to a longer term treatment with lower doses.

## 1 Introduction

Immune checkpoint inhibitors (ICI) is a type of immunotherapy that blocks membrane proteins, called checkpoints, on some T cells and cancer cells. It has revolutionized the treatment of various cancers, greatly prolonging the survival in advanced lung cancer, melanoma, and other cancers. However, severe side effects have emerged as a result of altering the natural immune response [1–3]. In particular, toxicity associated with ICI may result in pneumonitis, diarrhea, colitis, hepatitis, thyroid, and damage to the pituitary gland [1, 2, 4].

Steroids, or cortocosteroids, are anti-inflammatory drugs, frequently used in non-small cell lung cancer (NSCLC) and melanoma patients treated with ICI [5, 6]; they reduce toxicity, but also impair immunotherapy [7]. Steroids commonly used include prednisone and dexamethasone, that are known to decrease the number of cytotoxic lymphocytes [8, 9], hence also the concentration of inflammatory cytokines, such as IL-1, IL-6 and TNF-*α* [10]. Some studies show that using steroid too early after infusion of ICI decreases progression free survival (PFS) and overall survival (OS) [5, 11–13], but a recent review on metastatic analysis suggests that steroids, that are used only to mitigate adverse events of ICI, do not negatively affect overall survival [14]. The question of timing steroid initiation and response rates to ICI in metastatic cancer was considered in recent review by Maslov et al. [13]. Patients whose treatment with steroid began less than 2 months from ICI infusion had shorter PFS and OS than those who began treatment after more than 2 months.

CTLA-4 blockade, by ipilimumab, introduced in recent years in the treatment of metastatic melanoma, NSCLC and other cancers, incurs severe adverse events associated with toxicity, as observed by the high levels of TNF-*α* [15]. One of the most common immune related adverse events (irAEs) is hypophysitis [16–18]. Hypophysitis is a chronic inflammatory disease of the pituitary gland [19]. Since regulation of the pituitary function is controlled by cytokines [20], increased levels of inflammatory cytokines may disrupt the function of the gland. Hypophysitis can occur as autoimmune disease (primary), or as side-effect of treatment with ICI (secondary) [2]. Histological assessment identified T cells and dendritic cells (DCs) among the inflammatory cells in the pituitary gland of patients with hypophysitis [19, 21–24]. Secondary hypophysitis occurs in significant number of patients receiving ICI [19, 22, 24]. A form of hypophysitis is observed in more than 10% of metastatic melanoma and NSCLC patients receiving ipilimumab [16, 17, 25]. The mechanism involved in the targeted CTLA-4 vs. PD-1/PD-L1 in cancer treatment are not well understood [26]. It has been shown that CTLA-4 is expressed in normal pituitary gland cells [21] which could possibly explain why pituitary dysfunction is the most common immune-response adverse event of patients receiving CTLA-4 inhibitor therapy and not patients receiving PD-1/PD-L1 inhibitor [26, 27].

In what follows we take hypophysitis to represent all the irAEs. Indeed, hypophysitis occurs in significant larger percentage (10%) than any other severe adverse event: Palmeonitis occurs in 1.4% of patients undergoing immunotherapy with ICI [28], pneumonitis in 2.7% [29], myocarditis in 1% [30], neurologic diseases, including nephropathy, in 2.9% [31], colitis in 5.7–9.1% [32], and sustained acute kidney injury in 8% of patients [33]. Pancreatitis is negligible [34], and liver toxicity and cirrhosis are not common [35].

In this paper we consider an hypothetical treatment group of patients with metastatic cancer for which simulations are made about treatment with ICI in combination with steroid given in a specific schedule, and use a mathematical model to evaluate the comparative benefits of each schedule of steroid administration by determining both cancer growth and the risk of irAEs. More precisely, we consider a patient with NSCLC or metastatic melanoma and compute, for specific schedules of administration of prednisone, the tumor volume profile and the risk of hypophysitis as determined by the level of toxicity. The primary inflammatory cytokines are IL-1, IL-6 and TNF-*α*, but, since IL-1 and IL-6 are produced primarily by macrophages [36, 37], we simplify the model by introducing only TNF-*α* (which is produced by inflammatory CD4^+^ T cells [38]), and use the concentration of TNF-*α* to represent the level of toxicity. TNF-*α* diffuses from the tumor to the pituitary gland; an additional source of TNF-*α* originates in the pituitary gland as ipilimumab (which is infused into the blood) and targets the CTLA-4 expressed on pituitary cells. For simplicity, when considering levels of TNF-*α* below a prescribed threshold, we take the average concentration of TNF-*α* just within the tumor.

**Fig 1:**
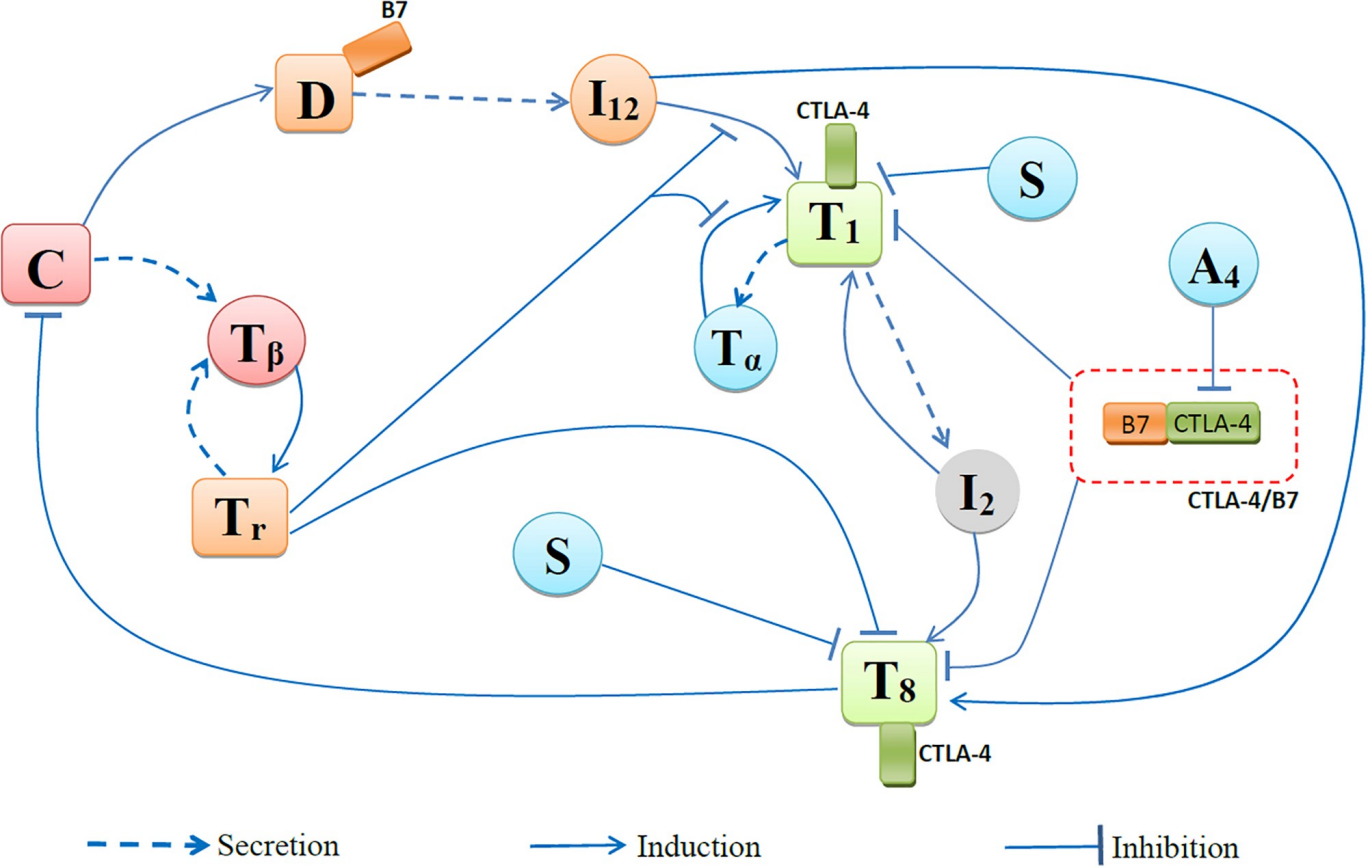
Network of cells and cytokines.

We develop a mathematical model based on the network in Fig 1. The model is represented by a system of partial differential equations, and is developed in a similar manner to other mathematical models of cancer-immune dynamics [39–46]. In [39], ICI was taken in combination with cancer vaccine, and, in [42], oncolytic virus therapy was supplemented by ICI; in [40], ICI was combined with BRAF/MEK inhibitor and in [41] it was combined with BET inhibitor, and combination of ICI with VEGF-inhibitor was considered in [43]. These papers addressed the questions what are effective protocols in terms of the amount of dose of each agent and what are optimal schedules of administration of the drugs. Other articles addressed the issue of drug resistance. In [44], it was shown that ICI reduces resistance to hormone therapy in metastatic prostate cancer; in [45] it was shown that resistance to ICI is reduced by anti-TNF-*α*; and in [46] it was shown that primary resistance to ICI can be overcome by anti-TGF-*β*. There are several mathematical models that represent treatment of cancer by ICI in terms of ordinary differential equations. A combination of ICI with immunostimulant was modeled in [47] by a system of 4 ODEs, and the effect of PD-L1 on tumor was studied in [48] using ODEs in three interrelated compartments; tumor, blood and spleen. A combination of ICI therapy with radiation was considered in [49] by a PDE system, and in [50] by a system of finite differences.

The species in the network in Fig 1 include cancer cells (*C*), CD8^+^ T cells (*T*_8_), CD4^+^ T cells (Th1 (*T*_1_) and Tregs (*T*_*r*_)), dendritic cells (*D*), and cytokines IL-2 (*I*_2_), IL-12 (*I*_12_), TGF-*β* (*T*_*β*_) and TNF-*α* (*T*_*α*_). The interactions among these species are represented by a system of partial differential equations (PDEs) in the tumor region Ω(*t*), which varies in time.

Dendritic cells (DCs), activated by cancer antigen, secrete IL-12, and IL-12 induces activation of naive CD4^+^ T cells and naive CD8^+^ T cells into *T*_1_ and *T*_8_ cells, respectively [51]. IL-2 is produced by *T*_1_, and it induces proliferation of both *T*_1_ and *T*_8_ [52, 53]. Cancer cells are killed primarily by *T*_8_ cells. The inflammatory TNF-*α* is produced by *T*_1_ cells and it activates them [38]. TGF-*β* is secreted by cancer cells [54] and by *T*_*r*_ [55], it induces proliferation of *T*_*r*_ cells [56], and *T*_*r*_ blocks the activation of both *T*_1_ and *T*_8_ cells [55, 57].

Dendritic cells express ligand B7, and the complex B7/CTLA-4 blocks the activation of naive T cells into *T*_1_ and *T*_8_. Prednisone (*S*) reduces toxicity by depleting *T*_1_ and *T*_8_ cells.

The mathematical model is based on representing the dynamics of these interactions by a system of partial differential equations.

## 2 Mathematical model

Table 1 lists all the variables of the model in units of g/cm^3^.

**Table 1:** Variables of the model. All densities and concentrations are in units of g/cm^3^.

Variables	Descriptions	Variables	Descriptions
*D*	density of dendritic cells	*T*_1_	density of Th1 cells
*T*_8_	density of CD8^+^ T cells	*T*_*r*_	density of Treg cells
*C*	density of cancer cells		
*I*_2_	concentration of IL-2	*I*_12_	concentration of IL-12
*T*_*α*_	concentration of TNF-*α*	*T*_*β*_	concentration of TGF-*β*
*B*_7_	concentration of B7	*P*_*A*_	concentration of CTLA-4
*Q*	concentration of B7/CTLA-4		
*A*_4_	concentration of anti-CTLA-4 (ipilimumab)	*S*	concentration of prednisone

We denote the tumor region, at time *t*, by Ω(*t*). This region varies with time and so are the cells within it. We assume that all the cells, as well as the cytokines, move with the same velocity **u**, and that they are also undergoing dispersion (diffusion).

The variables *X*_*i*_ satisfy a system of partial differential equations of the form

$$\begin{array}{c} \frac{\partial X_{i}}{\partial t}+\nabla \cdot \left(uX_{i}\right)-\delta_{X_{i}}\nabla^{2}X_{i}=F_{i}\left(X_{1},\ldots ,X_{n}\right)\;\text{in}\;\Omega (t),\;\left(i=1,\ldots ,n\right), \end{array}$$ (1)

where *F*_*i*_(*X*_1_, …, *X*_*n*_) is determined from the network of Fig 1, and $\delta_{X_{i}}$ is the diffusion coefficient.

### Equation for *D*

Immature dendritic cells (*D*_0_) are activated by HMGB-1 produced by necrotic cancer cells [58–60]. We represent this activation by a Michaelis-Menten rate λ_*DC*_*D*_0_*C*/(*K*_*C*_ + *C*), where *D*_0_ is the source of immature dendritic cells. Hence, the dynamics of DCs is given by

$$\frac{\partial D}{\partial t}+\nabla \cdot \left(uD\right)-\delta_{D}\nabla^{2}D=\lambda_{DC}D_{0}\frac{C}{K_{C}+C}-\mu_{D}D,$$ (2)

where *δ*_*D*_ is the diffusion coefficient and *μ*_*D*_ is the death rate.

### Equations for *T*_1_ and *T*_8_

Naive CD4^+^ T cells, *T*_10_, differentiate into *T*_1_ cells under *I*_12_ [51] and *T*_*α*_ [38] environment, a process inhibited by *T*_*r*_ [55]. The proliferation of activated *T*_1_ cells is enhanced by *I*_2_ [52, 53]. Both processes of activation and proliferation are inhibited by the complex B7/CTLA-4 (*Q*), by a factor 1/(1 + *Q*/*K*_*TQ*_), and prednisone (*S*) depletes *T*_1_ cells [8]. Hence, *T*_1_ satisfies the following equation:

$$\begin{array}{cc} \frac{\partial T_{1}}{\partial t} & \;+\nabla \cdot \left(uT_{1}\right)-\delta_{T_{1}}\nabla^{2}T_{1}=\\ & \;[T_{10}(\lambda_{T_{1}I_{12}}\frac{I_{12}}{K_{I_{12}}+I_{12}}+\lambda_{T_{1}T_{\alpha }}\frac{T_{\alpha }}{K_{T_{\alpha }}+T_{\alpha }})\frac{1}{1+T_{r}/K_{TT_{r}}}+\lambda_{T_{1}I_{2}}T_{1}\frac{I_{2}}{K_{I_{2}}+I_{2}}]\frac{1}{1+Q/K_{TQ}}\\ \; & -\mu_{T_{1}}T_{1}-\mu_{ST}ST_{1}. \end{array}$$ (3)

Similarly,

$$\begin{array}{cc} \frac{\partial T_{8}}{\partial t} & \;+\nabla \cdot \left(uT_{8}\right)-\delta_{T_{8}}\nabla^{2}T_{8}=\\ & \;[T_{80}(\lambda_{T_{8}I_{12}}\frac{I_{12}}{K_{I_{12}}+I_{12}}+\lambda_{T_{8}T_{\alpha }}\frac{T_{\alpha }}{K_{T_{\alpha }}+T_{\alpha }})\frac{1}{1+T_{r}/K_{TT_{r}}}+\lambda_{T_{8}I_{2}}T_{8}\frac{I_{2}}{K_{I_{2}}+I_{2}}]\frac{1}{1+Q/K_{TQ}}\\ \; & -\mu_{T_{8}}T_{8}-\mu_{ST}ST_{8}; \end{array}$$ (4)

note that *T*_*r*_ also controls the activation of *T*_8_ cells [57].

### Equation for *T*_*r*_

The activation of *T*_*r*_ is induced by TGF-*β* [56], so that

$$\frac{\partial T_{r}}{\partial t}+\nabla \cdot \left(uT_{r}\right)-\delta_{T_{r}}\nabla^{2}T_{r}=\lambda_{T_{r}T_{\beta }}T_{10}\frac{T_{\beta }}{K_{T_{\beta }}+T_{\beta }}-\mu_{T_{r}}T_{r}.$$ (5)

### Equation for *C*

We assume logistic growth of cancer cells, with carrying capacity *C*_*M*_, to account for their competition for space and nutrients. Cancer cells are killed primarily by CD8^+^ T cells, hence

$$\frac{\partial C}{\partial t}+\nabla \cdot \left(uC\right)-\delta_{C}\nabla^{2}C=\lambda_{C}C(1-\frac{C}{C_{M}})-\mu_{T_{8}C}T_{8}C-\mu_{C}C,$$ (6)

where *μ*_*C*_ is the death rate of cancer cells, and *μ*_*T*_8_*C*_ is the rate by which *T*_8_ cells kill cancer cells. As the network in Fig 1 shows, cancer cells try to block *T*_8_ cells, but dendritic cells, activated by cancer antigen, lead to proliferation of *T*_8_ cells; CTLA-4 inhibitor (*A*_4_) increases the activation of *T*_8_, and steroid (*S*) decreases it while reducing toxicity.

In order to determine the velocity **u**, we need a constitutional assumption on the tumor tissue. For simplicity, we consider only the case of a spherically symmetric tumor and spherically symmetric variables. Then **u** = *u* **e**_*r*_ where **e**_*r*_ is the unit radial vector, and Ω(*t*) = {0 ≤ *r* ≤ *R*(*t*)} is a sphere with boundary *r* = *R*(*t*). In order to determine *u*(*r*, *t*) we assume the combined density of all the cells in the tumor region Ω(*t*) remains constant in space and time, so that

$$\begin{array}{c} D+T_{1}+T_{8}+T_{r}+C=\text{const}. \end{array}$$ (7)

We then add all the cell equations and assume that their diffusion coefficients are approximately equal. Using Eq (7), we then obtain the following equation for **u**:

$$\begin{array}{c} \frac{1}{r^{2}}\frac{\partial }{\partial r}\left(r^{2}u\right)=\nabla \cdot u=\text{const}\cdot \Sigma \left\{\text{Right-hand-side}\;\text{of}\;\text{all}\;\text{cell}\;\text{equations}\right\}, \end{array}$$ (8)

with *u*(0, *t*) = 0.

We assume that the boundary of Ω(*t*) varies with the velocity **u**, hence

$$\begin{array}{c} \frac{dR(t)}{dt}=u\left(R\left(t\right),t\right). \end{array}$$

### Equations for cytokines

IL-2 is produced by *T*_1_ cells [52, 53], so that

$$\frac{\partial I_{2}}{\partial t}-\delta_{I_{2}}\nabla^{2}I_{2}=\lambda_{I_{2}T_{1}}T_{1}-\mu_{I_{2}}I_{2},$$ (9)

where $\mu_{I_{2}}$ is a degradation rate. Note that the diffusion coefficient of cytokines is several orders of magnitude larger than the diffusion coefficient of cells, hence their advection velocity is negligible relative to their diffusion, and may therefore be dropped.

IL-12 is secreted by DCs [61, 62]. IL-12 is also secreted by iosinophils as a result of toxicity due to immunotherapy [63], which we assume to be proportional to *A*_4_. Activated Th1 cells are the main receptors of IL-12, which means that they decrease IL-12 ligands in the process of being activated [64]. Hence,

$$\frac{\partial I_{12}}{\partial t}-\delta_{I_{12}}\nabla^{2}I_{12}=\lambda_{I_{12}D}D+\lambda_{I_{12}A_{4}}A_{4}-\mu_{I_{12}T_{1}}T_{1}\frac{I_{12}}{K_{I_{12}}+I_{12}}-\mu_{I_{12}}I_{12},$$ (10)

where $\mu_{I_{12}}$ is a degradation rate.

TNF-*α* is produced by *T*_1_ cells [38], so that

$$\frac{\partial T_{\alpha }}{\partial t}-\delta_{T_{\alpha }}\nabla^{2}T_{\alpha }=\lambda_{T_{\alpha }T_{1}}T_{1}-\mu_{T_{\alpha }}T_{\alpha }.$$ (11)

TGF-*β* is produced by cancer cells [54] and *T*_*r*_ cells [55], hence

$$\frac{\partial T_{\beta }}{\partial t}-\delta_{T_{\beta }}\nabla^{2}T_{\beta }=\lambda_{T_{\beta }C}C+\lambda_{T_{\beta }T_{r}}T_{r}-\mu_{T_{\beta }}T_{\beta }.$$ (12)

### Equation for B7 (*B*_7_), CTLA-4 (*P*_*A*_) and B7/CTLA-4 (*Q*)

CTLA-4 is a receptor expressed on activated *T*_1_ and *T*_8_ cells [65] and the complex B7/CTLA-4 blocks the activities of these cells [65, 66]. CTLA-4 is constitutively expressed on *T*_*r*_ cells, but its activity is not blocked by the complex B7/CTLA-4 [67]. We assume that the number of CTLA-4 proteins per cell is the same for *T*_1_ and *T*_8_ cells, but different for *T*_*r*_ cells, by a factor *κ*_*T*_. We denote by $\rho_{P_{A}}$ the ratio between the mass of all CTLA-4 proteins in one T cell to the mass of this cell, so that

$$\begin{array}{c} P_{A}=\rho_{P_{A}}\left(T_{1}+T_{8}+\kappa_{T}T_{r}\right). \end{array}$$

The coefficient $\rho_{P_{A}}$ is constant when no anti-CTLA-4 drug is administered. In this case, to a change in *T* (*T*_1_, *T*_8_, *T*_*r*_), given by ∂*T*/∂*t*, there corresponds a change of *P*_*A*_, given by $\rho_{P_{A}}\partial T/\partial t$. Similar changes in *P*_*A*_ arises from the terms of diffusion and advection, so that

$$\begin{array}{cc} \frac{\partial P_{A}}{\partial t}+ & \nabla \cdot \left(uP_{A}\right)-\delta_{T}\nabla^{2}P_{A}=\\ & \rho_{P_{A}}\{\;[(\left(\lambda_{T_{1}I_{12}}T_{10}+\lambda_{T_{8}I_{12}}T_{80}\right)\frac{I_{12}}{K_{I_{12}}+I_{12}}+\left(\lambda_{T_{1}T_{\alpha }}T_{10}+\lambda_{T_{8}T_{\alpha }}T_{80}\right)\frac{T_{\alpha }}{K_{T_{\alpha }}+T_{\alpha }})\frac{1}{1+T_{r}/K_{TT_{r}}}\\ & +\left(\lambda_{T_{1}I_{2}}T_{1}+\lambda_{T_{8}I_{2}}T_{8}\right)\frac{I_{2}}{K_{I_{2}}+I_{2}}]\frac{1}{1+Q/K_{TQ}}\\ & +\kappa_{T}\lambda_{T_{r}T_{\beta }}T_{10}\frac{T_{\beta }}{K_{T_{\beta }}+T_{\beta }}-\left(\mu_{T_{1}}T_{1}+\mu_{T_{8}}T_{8}+\mu_{ST}S\left(T_{1}+T_{8}\right)+\kappa_{T}\mu_{T_{r}}T_{r}\right)\;\}. \end{array}$$

When anti-CTLA-4 drug (*A*_4_) is applied, CTLA-4 is depleted at a rate proportional to *A*_4_, and, in this case, the ratio *P*_*A*_/(*T*_1_ + *T*_8_ + *κ*_*T*_*T*_*r*_) may change. In order to include in the model both cases, with and without anti-CTLA-4, we replace $\rho_{P_{A}}$ in the above equation by *P*_*A*_/(*T*_1_ + *T*_8_ + *κ*_*T*_*T*_*r*_). Hence,

$$\begin{array}{cc} \frac{\partial P_{A}}{\partial t}+ & \nabla \cdot \left(uP_{A}\right)-\delta_{T}\nabla^{2}P_{A}=\frac{P_{A}}{\left(T_{1}+T_{8}+\kappa_{T}T_{r}\right)}\\ & \times \{\;[(\left(\lambda_{T_{1}I_{12}}T_{10}+\lambda_{T_{8}I_{12}}T_{80}\right)\frac{I_{12}}{K_{I_{12}}+I_{12}}+\left(\lambda_{T_{1}T_{\alpha }}T_{10}+\lambda_{T_{8}T_{\alpha }}T_{80}\right)\frac{T_{\alpha }}{K_{T_{\alpha }}+T_{\alpha }})\frac{1}{1+T_{r}/K_{TT_{r}}}\\ & +\left(\lambda_{T_{1}I_{2}}T_{1}+\lambda_{T_{8}I_{2}}T_{8}\right)\frac{I_{2}}{K_{I_{2}}+I_{2}}]\frac{1}{1+Q/K_{TQ}}\\ & +\kappa_{T}\lambda_{T_{r}T_{\beta }}T_{10}\frac{T_{\beta }}{K_{T_{\beta }}+T_{\beta }}-\left(\mu_{T_{1}}T_{1}+\mu_{T_{8}}T_{8}+\mu_{ST}S\left(T_{1}+T_{8}\right)+\kappa_{T}\mu_{T_{r}}T_{r}\right)\;\}-\mu_{P_{A}A_{4}}P_{A}A_{4}, \end{array}$$ (13)

where $\mu_{P_{A}A_{4}}$ is the depletion rate of CTLA-4 by anti-CTLA-4.

Ligand B7 is expressed on dendritic cells, so that

$$B_{7}=\rho_{B_{7}}D,\;\rho_{B_{7}}=\text{constant}.$$

B7 and CTLA-4 from the complex B7/CTLA-4 (*Q*) with association and disassociation rates $\alpha_{B_{7}P_{A}}$ and *μ*_*Q*_, respectively:

$$\begin{array}{c} B_{7}+P_{A}\underset{\mu_{Q}}{\overset{\alpha_{B_{7}P_{A}}}{⇌}}Q. \end{array}$$

We assume that the half-life of *Q* is very short [68, 69], so that we may approximate the dynamics of *Q* by the steady state, $\alpha_{B_{7}P_{A}}B_{7}P_{A}=\mu_{Q}Q$, or

$$\begin{array}{c} Q=\sigma B_{7}P_{A}, \end{array}$$ (14)

where $\sigma =\alpha_{B_{7}P_{A}}/\mu_{Q}$.

### Equations for *A*_4_ and *S*

The concentration of anti-CTLA-4 satisfies the equation

$$\begin{array}{c} \frac{\partial A_{4}}{\partial t}-\delta_{A_{4}}\nabla^{2}A_{4}=\underset{\text{source}}{\underset{︸}{\gamma_{A_{4}}f_{A_{4}}\left(t\right)}}-\underset{\text{depletion}\;\text{through}\;\text{blocking}\;\text{CTLA-4}}{\underset{︸}{\mu_{P_{A}A_{4}}P_{A}A_{4}}}-\underset{\text{degradation}}{\underset{︸}{\mu_{A_{4}}A_{4}}} \end{array}$$ (15)

where the drug is injected at dose $\gamma_{A_{4}}$ several times during treatment, and its actual strength at time *t* is $\gamma_{A_{4}}f_{A_{4}}\left(t\right)$.

Similarly, the concentration of prednisone satisfies the following equation:

$$\begin{array}{c} \frac{\partial S}{\partial t}-\delta_{S}\nabla^{2}S=\underset{\text{source}}{\underset{︸}{\gamma_{S}f_{S}\left(t\right)}}-\underset{\text{depletion}\;\text{through}\;\text{blocking}\;T_{1}\;\text{and}\;T_{8}}{\underset{︸}{\mu_{TS}\left(T_{1}+T_{8}\right)S}}-\underset{\text{degradation}}{\underset{︸}{\mu_{S}S}}. \end{array}$$ (16)

### Boundary conditions

We assume that the tumor boundary, ∂Ω(*t*), is moving with velocity of the cells, that is

$$\begin{array}{c} V_{n}=u\cdot n \end{array}$$ (17)

where **n** is the outward normal at boundary and *V*_*n*_ is the velocity of the free boundary of the tumor in the direction **n**.

We assume that the inactive CD4^+^ and CD8^+^ T cells that migrated from the lymph nodes into the tumor microenvironment have constant densities ${\hat{T}}_{1}$ and ${\hat{T}}_{8}$, respectively, at the tumor boundary, and that they are activated by IL-12 upon entering the tumor. We then have the following conditions at the tumor boundary:

$$\begin{array}{cc} \frac{\partial T_{1}}{\partial t} & +\sigma_{0}\frac{I_{12}}{K_{I_{12}}+I_{12}}{\left(T_{1}-{\hat{T}}_{1}\right)}^{+}=0,\\ \frac{\partial T_{8}}{\partial r} & +\sigma_{0}\frac{I_{12}}{K_{I_{12}}+I_{12}}{\left(T_{8}-{\hat{T}}_{8}\right)}^{+}=0\;\text{at}\;r=R(t). \end{array}$$ (18)

We impose no-flux boundary condition on all the remaining variables:

$$\begin{array}{c} \text{No}\;\text{flux}\;\text{for}\;C,\;T_{r},\;D,\;I_{2},\;I_{12},\;T_{\alpha },\;T_{\beta },\;P_{A},\;A_{4},\;\text{and}\;S\;\text{at}\;r=R\left(t\right); \end{array}$$ (19)

it is tacitly assumed here that CTLA-4 become actives only after the T cells are already inside the tumor.

We prescribe initial conditions (in units of g/cm^3^) after some time when the tumor was already established and the immune cells are activated:

$$\begin{array}{cc} C= & 0.41,\;T_{1}=10^{-3},\;T_{8}=2.4\times 10^{-4},\;T_{r}=10^{-5},\;D=10^{-6},\\ I_{2}= & 1.5\times 10^{-11},\;I_{12}=5\times 10^{-11},\;T_{\alpha }=4.4\times 10^{-12},\;T_{\beta }=7.4\times 10^{-9},\;\text{and}\;R=0.13\;\text{cm}. \end{array}$$ (20)

Other nearby choices of initial conditions do not affect the simulations of the model after a few days.

## 3 Results

All the computations were done using Python 3.5.4. The parameter values of the model equations, except $\mu_{TS},\;\mu_{ST},\;\mu_{P_{A}A_{4}}$ and *C*_*M*_, are estimated in Supporting Information and are listed in Tables 2 and 3. Parameter sensitivity analysis was performed using Latin Hypercube Sampling/Partial Rank Correlation Coefficient (LHS/PRCC) and presented in the Supporting Information, and the numerical scheme used in the simulations, the moving mesh method, is also described in the Supporting Information.

**Table 2:** Parameters for the model.

Parameters	Descriptions	Values	references
λ_*C*_	proliferation rate of *C*	0.203 d^−1^	[75] est.
*C*_*M*_	carrying capacity for *N* and *M*	4.9 g/cm^3^	fitted
*D*_0_	source of *D*	2 × 10^−5^ g/cm^3^	[41]
*T*_10_	source of *T*_1_	4 × 10^−4^ g/cm^3^	[41]
*T*_80_	source of *T*_8_	2 × 10^−4^ g/cm^3^	[41]
${\hat{T}}_{1}$	inflow of *T*_1_ from lymph node	4 × 10^−3^ g/cm^3^	[41]
${\hat{T}}_{8}$	inflow of *T*_8_ from lymph node	2 × 10^−3^ g/cm^3^	[41]
*μ*_*C*_	rate of death of *C*	0.17 d^−1^	[76] est.
*μ*_*D*_	rate of death of *D*	0.13 d^−1^	[77] est.
$\mu_{T_{1}}$	rate of death of *T*_1_	0.2 d^−1^	[78] est.
$\mu_{T_{8}}$	rate of death of *T*_8_	0.2 d^−1^	[78] est.
$\mu_{T_{r}}$	rate of death of *T*_*r*_	0.25 d^−1^	[79] est.
$\mu_{I_{2}}$	rate of decay of *I*_2_	166.22 d^−1^	[80] est.
$\mu_{I_{12}}$	rate of decay of *I*_12_	2.13 d^−1^	[81] est.
$\mu_{T_{\alpha }}$	rate of decay of *T*_*α*_	199 d^−1^	[82, 83] est.
$\mu_{T_{\beta }}$	rate of decay of *T*_*β*_	399.25 d^−1^	[46]
$\mu_{A_{4}}$	decay rate of *A*_4_	4.72 × 10^−2^ d^−1^	[84] est.
*μ*_*S*_	decay rate of *S*	4.62/5.54 d^−1^	[73, 85] est.
*μ*_*Q*_	decay rate of *Q*	6 × 10^4^ d^−1^	[68]
*δ*_*C*_, *δ*_*D*_, *δ*_*T*_	diffusion coefficient of cells	8.64 × 10^−7^ cm^2^d^−1^	[41] est.
$\delta_{I_{2}}$	diffusion coefficient of *I*_2_	9.92 × 10^−2^ cm^2^d^−1^	[86, 87] est.
$\delta_{I_{12}}$	diffusion coefficient of *I*_12_	7.5 × 10^−2^ cm^2^d^−1^	[86, 87] est.
$\delta_{T_{\alpha }}$	diffusion coefficient of *T*_*α*_	9.76 × 10^−2^ cm^2^d^−1^	[86, 88] est.
$\delta_{T_{\beta }}$	diffusion coefficient of *T*_*β*_	14.86 × 10^−2^ cm^2^d^−1^	[86, 89] est.
$\delta_{A_{4}}$	diffusion coefficient of *A*_4_	7.5 × 10^−2^ cm^2^d^−1^	[86, 90] est.
*δ*_*S*_	diffusion coefficient of *S*	3.51 × 10^−2^ cm^2^d^−1^	[86, 91] est.
$\mu_{T_{8}C}$	killing rate of *C* by *T*_8_	33 cm^3^/g⋅d	est.
$\mu_{P_{A}A_{4}}$	rate of depletion of *A*_4_ by *P*_*A*_	1.1 × 10^7^ cm^3^/g⋅d	fitted
*μ*_*ST*_	inhibition rate of *T*_1_ and *T*_8_ by *S*	5.31 × 10^6^ cm^3^/g⋅d	fitted
*μ*_*TS*_	absorption rate of *S* by *T*_1_ and *T*_8_	9 × 10^3^ cm^3^/g⋅d	fitted
$\mu_{I_{12}T_{1}}$	decay of *I*_12_ due to *T*_1_ and *T*_8_	10^−7^ cm^3^/g⋅d	fitted

est.= this parameter was estimated in Supporting Information.

**Table 3:** Parameters for the model (continued).

Parameters	Descriptions	Values	references
λ_*DC*_	activation rate of *D* by *C*	5.2 d^−1^	est.
$\lambda_{T_{1}I_{2}}$	proliferation rate of *T*_1_ by *I*_2_	0.25 d^−1^	[41]
$\lambda_{T_{8}I_{2}}$	proliferation rate of *T*_8_ by *I*_2_	0.25 d^−1^	[41]
$\lambda_{T_{1}I_{12}}$	activation rate of *T*_1_ by *I*_12_	1.375 d^−1^	est.
$\lambda_{T_{8}I_{12}}$	activation rate of *T*_8_ by *I*_12_	1.375 d^−1^	est.
$\lambda_{T_{1}T_{\alpha }}$	activation rate of *T*_1_ by *T*_*α*_	4.125 d^−1^	est.
$\lambda_{T_{8}T_{\alpha }}$	activation rate of *T*_8_ by *T*_*α*_	1.375 d^−1^	est.
$\lambda_{T_{r}T_{\beta }}$	activation rate of *T*_*r*_ by *T*_*β*_	0.13 d^−1^	est.
$\lambda_{I_{2}T_{1}}$	production rate of *I*_2_ by *T*_1_	1.6 × 10^−6^ d^−1^	est.
$\lambda_{I_{12}D}$	production rate of *I*_12_ by *D*	3.03 × 10^−6^ d^−1^	est.
$\lambda_{I_{12}A_{4}}$	production rate of *I*_12_ due to *A*_4_	10^−3^ d^−1^	est.
$\lambda_{T_{\alpha }T_{1}}$	production rate of *T*_*α*_ by *T*_1_	8.4 × 10^−7^ d^−1^	fitted
$\lambda_{T_{\beta }C}$	production rate of *T*_*β*_ by *C*	7.2 × 10^−6^ d^−1^	est.
$\lambda_{T_{\beta }T_{r}}$	production rate of *T*_*β*_ by *T*_*r*_	3.6 × 10^−6^ d^−1^	est.
*K*_*D*_	half saturation of *D*	4 × 10^−4^ g/cm^3^	[41]
$K_{TT_{r}}$	inhibition of *T*_1_ and *T*_8_ by *T*_*r*_	1.04 × 10^−4^ g/cm^3^	[92] est.
$K_{I_{2}}$	half saturation of *I*_2_	1.9 × 10^−11^ g/cm^3^	[93] est.
$K_{I_{12}}$	half saturation of *I*_12_	10^−10^ g/cm^3^	[93] est.
$K_{T_{\alpha }}$	half saturation of *T*_*α*_	8.4 × 10^−12^ g/cm^3^	[88] est.
$K_{T_{\beta }}$	half saturation of *T*_*β*_	7.2 × 10^−9^ g/cm^3^	[46]
*K*_*Q*_	half saturation of *Q*	4.86 × 10^−20^ g^2^/cm^6^	[41]
$K_{TQ}^{\prime }$	inhibition of *T*_1_ and *T*_8_ by *P*_*A*_-*B*_7_	4.86 × 10^−20^ g^2^/cm^6^	[41]
*θ*	constant density of cells	0.5 g/cm^3^	est.
*κ*_*T*_	(#*P*_*A*_ per *T*_*r*_)/(#*P*_*A*_ per *T*_1_ or *T*_8_)	1	est.

est.= this parameter was estimated in Supporting Information.

The simulations are carried out in the case of radially symmetric tumor, where Ω(*t*) = {0 ≤ *r* ≤ *R*(*t*)}, and radially symmetric variables, that is, functions of (*r*, *t*), where *r* = |*x*| is the distance of a point *x* to the origin, and **u** = *u* **e**_*r*_ where *u* = *u*(*r*, *t*) and **e**_*r*_ is the unit vector *x*/|*x*|. Then Eq (17) becomes

$$\begin{array}{c} \frac{dR(t)}{dt}=u\left(R\left(t\right),t\right). \end{array}$$ (21)

From Eq (7) we then deduce that

$$\begin{array}{c} \frac{dR(t)}{dt}=\frac{\theta }{R^{2}\left(t\right)}\int_{0}^{R(t)}[\sum_{j=2}^{6}\text{R.H.S.}\;\text{of}\;\text{Eqs}.\;(2.\text{j})]r^{2}dr. \end{array}$$ (22)

### Mice experiments

In order to determine the effect of steroid on the efficacy of ipilimumab, we use some data from mice experiments by Tokunaga et al. [70] and Giles et al. [9]. We assume that a drug *A*, with half-life *m*, injected at time *t* = 0 with dose *A*_0_, degrades exponentially, so that

$$\begin{array}{c} \frac{dA}{dt}=-A_{0}e^{-\mu_{A}t}, \end{array}$$

where

$$\begin{array}{c} \mu_{A}=\frac{\text{ln}\;2}{m}. \end{array}$$

In the experiments in [70] Figs 1A, 1B and 3H (for convenience we have added this figure in the S1 Appendix), *A*_4_ was injected in days 0, 3, 6 and *S* was injected in days 3, 5, 7 with low dose *γ*_*S*_, and high dose 100*γ*_*S*_; the end-time was day 40. In the corresponding Eqs (15) and (16) we then have,

$$\begin{array}{c} f_{A_{4}}\left(t\right)=\{\begin{array}{cc} e^{-\mu_{A_{4}}t}, & \text{for}\;0\leq t<3,\\ e^{-\mu_{A_{4}}t}+e^{-\mu_{A_{4}}\left(t-3\right)}, & \text{for}\;3\leq t<6,\\ e^{-\mu_{A_{4}}t}+e^{-\mu_{A_{4}}\left(t-3\right)}+e^{-\mu_{A_{4}}\left(t-6\right)}, & \text{for}\;t\geq 6, \end{array}\\ f_{S}\left(t\right)=\{\begin{array}{cc} e^{-\mu_{S}\left(t-3\right)}, & \text{for}\;3\leq t<5,\\ e^{-\mu_{S}\left(t-3\right)}+e^{-\mu_{S}\left(t-5\right)}, & \text{for}\;5\leq t<7,\\ e^{-\mu_{S}\left(t-3\right)}+e^{-\mu_{S}\left(t-5\right)}+e^{-\mu_{S}\left(t-7\right)}, & \text{for}\;t\geq 7. \end{array} \end{array}$$

The steroid used in [70] is methylprednisolone, which is slightly stronger than prednisone, but has a slightly shorter half-life.

**Fig 2:**
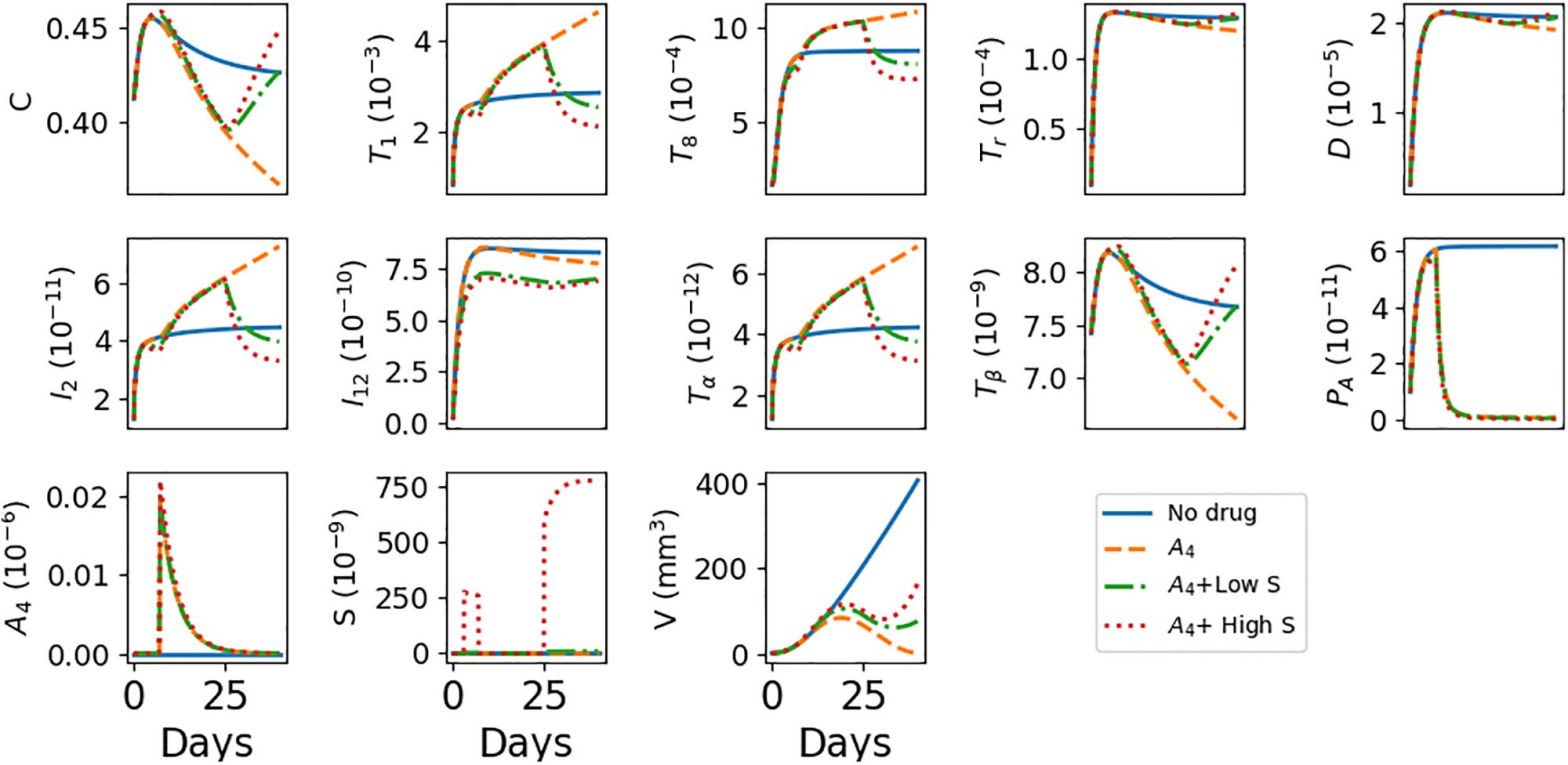
Simulation of the average densities/concentrations of the variables with/without anti-CTLA-4 and prednisone at $\gamma_{A_{4}}=2\times 10^{-8}$ g/cm^3^⋅d, *γ*_*S*_ = 7 × 10^−9^ g/cm^3^⋅d (low) and *γ*_*S*_ = 700 × 10^−9^ g/cm^3^⋅d (high). All the model variables, with various drug combinations. All parameters are as in Tables 1 and 2 of Supplementary Information. *A*_4_ alone reduces tumor growth but increases TNF-*α*, while *A*_4_, when combined with *S*, reduces TNF-*α* but increases tumor growth. All species are in units of g/cm^3^.

Fig 2 shows the average densities/concentrations of all model’s variables and the tumor volume during 40 days, *A*_4_ as single agent, with *A*_4_+low *γ*_*S*_, and *A*_4_+high *γ*_*S*_, where $\gamma_{A_{4}}=2\times 10^{-8}$ g/cm^3^⋅d, low *γ*_*S*_ = 7 × 10^−9^ g/cm^3^⋅d, and high *γ*_*S*_ = 700 × 10^−9^ g/cm^3^⋅d. Note that in the no-drug case, the slow increase in *T*_*α*_ is associated with the slow increase in *T*_1_.

**Fig 3:**
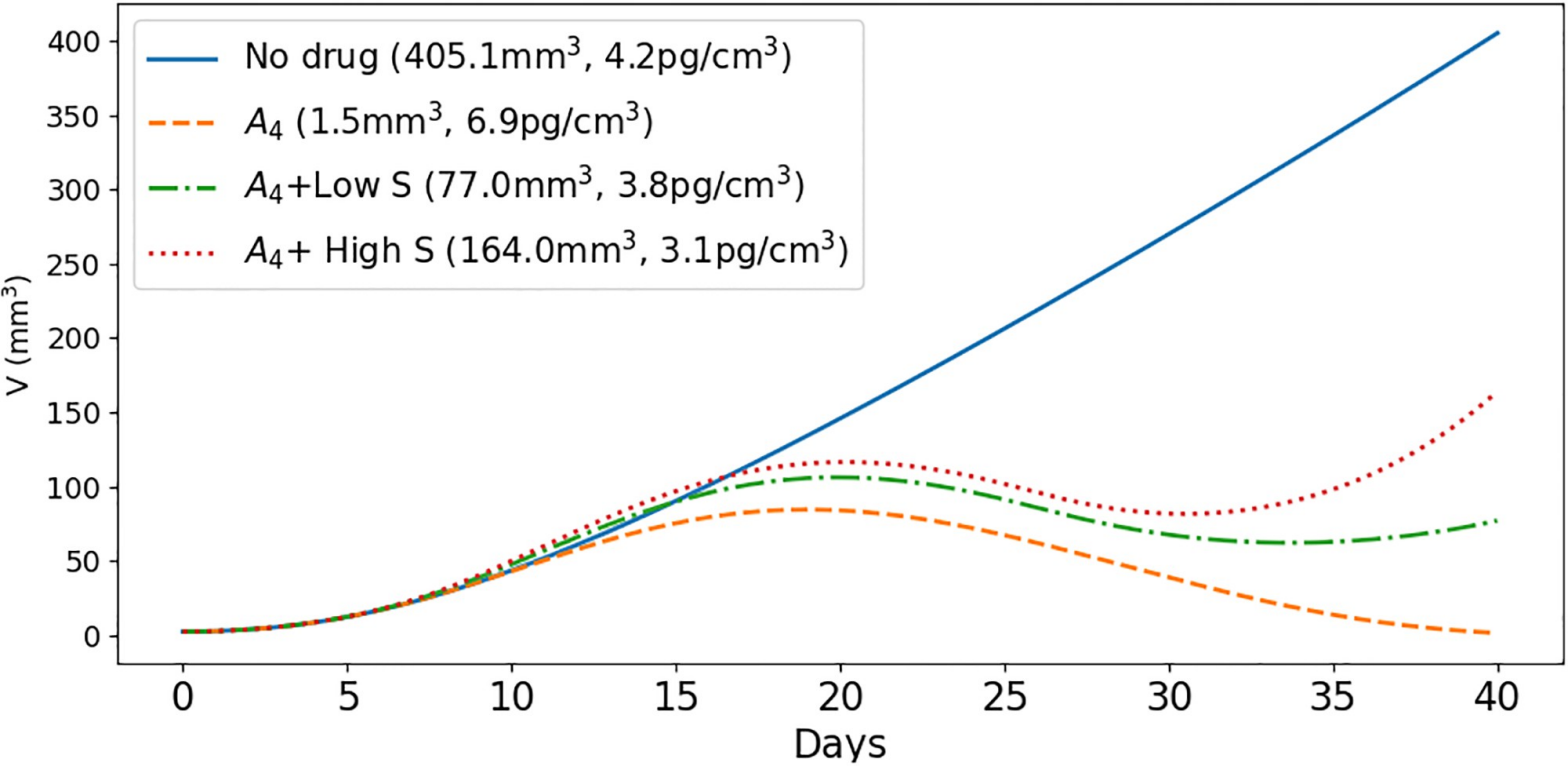
Simulation of the average densities/concentrations of the variables with/without anti-CTLA-4 and prednisone at $\gamma_{A_{4}}=2\times 10^{-8}$ g/cm^3^⋅d, *γ*_*S*_ = 7 × 10^−9^ g/cm^3^⋅d (low) and *γ*_*S*_ = 700 × 10^−9^ g/cm^3^⋅d (high). Tumor volume only in various treatment combinations. All parameters are as in Tables 1 and 2 of Supplementary Information. *A*_4_ alone reduces tumor growth but increases TNF-*α*, while *A*_4_, when combined with *S*, reduces TNF-*α* but increases tumor growth. The pair (*, *) represents the tumor volume and the concentration of TNF-*α* at day 40. All species are in units of g/cm^3^.

Fig 3 shows, more clearly, the profiles of tumor volume as they evolve from day to day. We see that, under treatment with *A*_4_, the initial rate of increase of tumor volume begins to decrease around day 12 and to slowly change from increasing to decreasing, until it reaches the initial volume around day 40, where it shows tendency to slightly begin increasing.

Under *A*_4_+low *S*, the volume profile is similar to that of the case of *A*_4_, but the initial phases of increase and slowly changing to decrease end around day 32, and then the volume is continuously increasing. The volume profile under *A*_4_+high *S* is similar, but the final increase occurs earlier, in day 28, and we see a significant increase by day 40 compared to the case of *A*_4_+low *S*. We also note that, under *A*_4_+high *S*, tumor volume exceeds the control case during days 11–17.

All the above features are the same as in [70] Fig 1A and 1B, except that in [70] the changes from increase to decrease to increase occur earlier. This discrepancy may be explained by our choice of initial conditions which are not fitted to the actual experiment. We note that unlike methylprednisolone used in [70], prednisone, that will be used in our clinical trials, must first be converted in the liver into enzyme.

**Fig 4:**
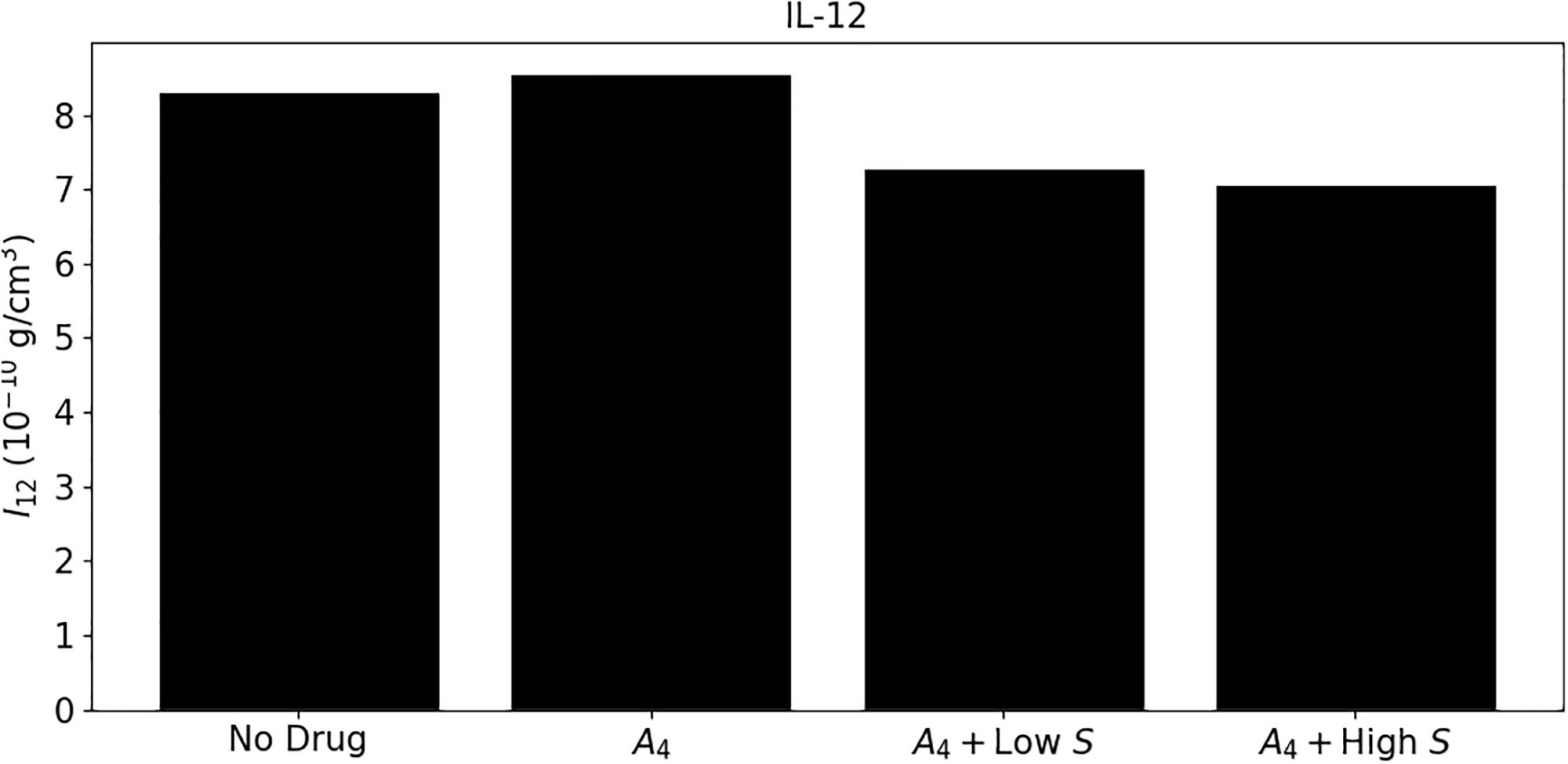
Levels of IL-12 with/without anti-CTLA-4 and prednisone at $\gamma_{A_{4}}=2\times 10^{-8}$ g/cm^3^⋅d, *γ*_*S*_ = 7 × 10^−9^ g/cm^3^⋅d (low) and *γ*_*S*_ = 700 × 10^−9^ g/cm^3^⋅d (high).

In Fig 4 we simulated the levels of IL-12 at day 40 in the cases of no-drug, *A*_4_+low *S*, *A*_4_+high *S*, and the maximum level attained by IL-12 in the course of treatment with *A*_4_ alone, which occurs right after day 7 when the full dose of *A*_4_ is given (note that in [70] Fig 3H the level of IL-12 was measured at day 10 right after the full dose of *A*_4_ was given). We see that the level of IL-12 under *A*_4_ is above the level of the control case (no drugs), and it decreases under *A*_4_+low *S* to below the level of the control case; it further decreases under *A*_4_+high *S*. This pattern is in agreement with [70] Fig 3H; although Fig 3H deals with the case of low affinity among CD8^+^ T cells, this does not significantly affect dendritic cells and *T*_1_ cells, and hence it also does not significantly affect IL-12 (by Eq (10)).

**Fig 5:**
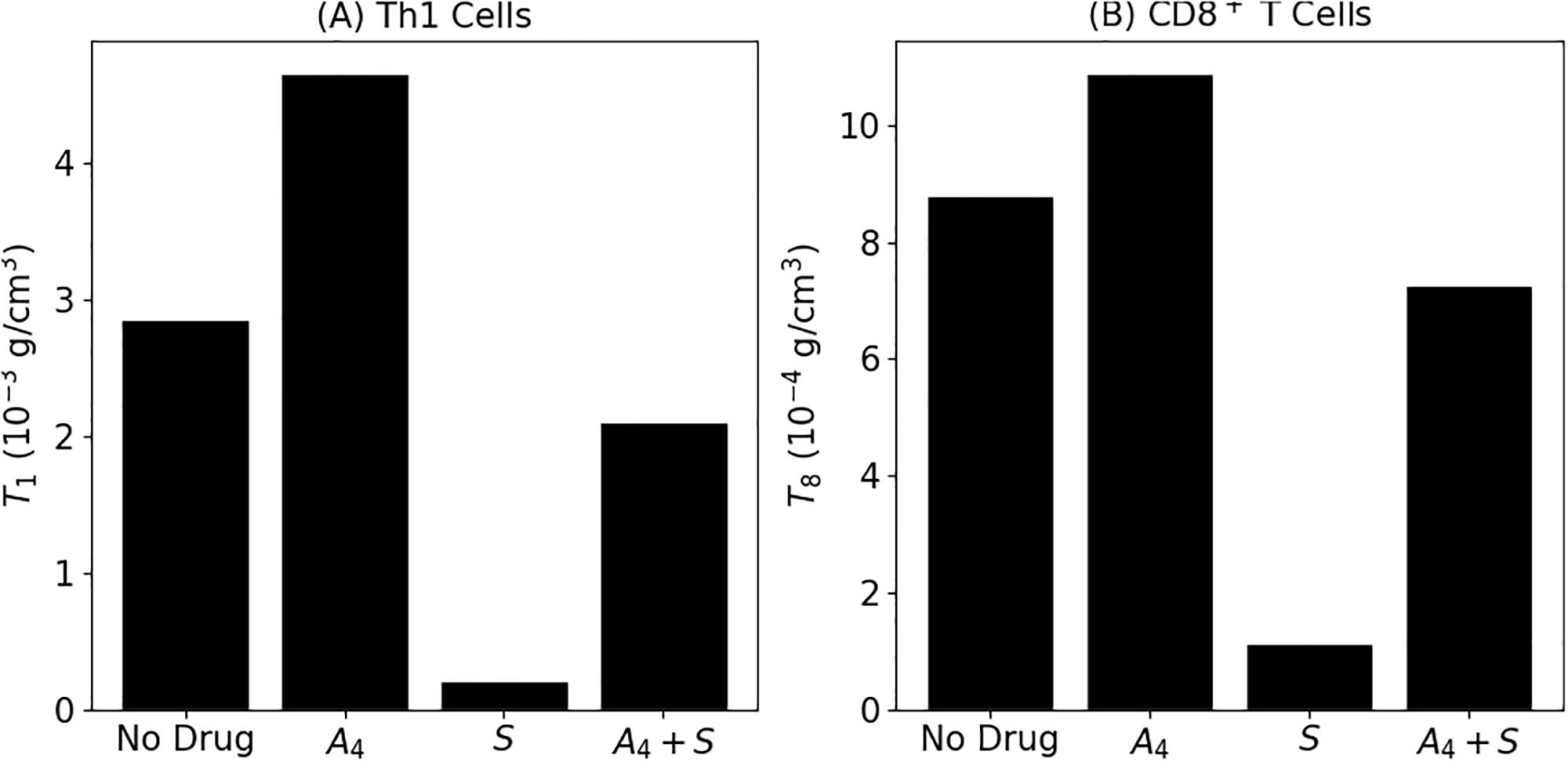
T cell levels with/without anti-CTLA-4 and prednisone at $\gamma_{A_{4}}=2\times 10^{-8}$ g/cm^3^⋅d, *γ*_*S*_ = 700 × 10^−9^ g/cm^3^⋅d (high) and *γ*_*S*_ = 7000 × 10^−9^ g/cm^3^⋅d (alone). Density of Th1 cells (A) and density of CD8^+^ T cells (B).

**Fig 6:**
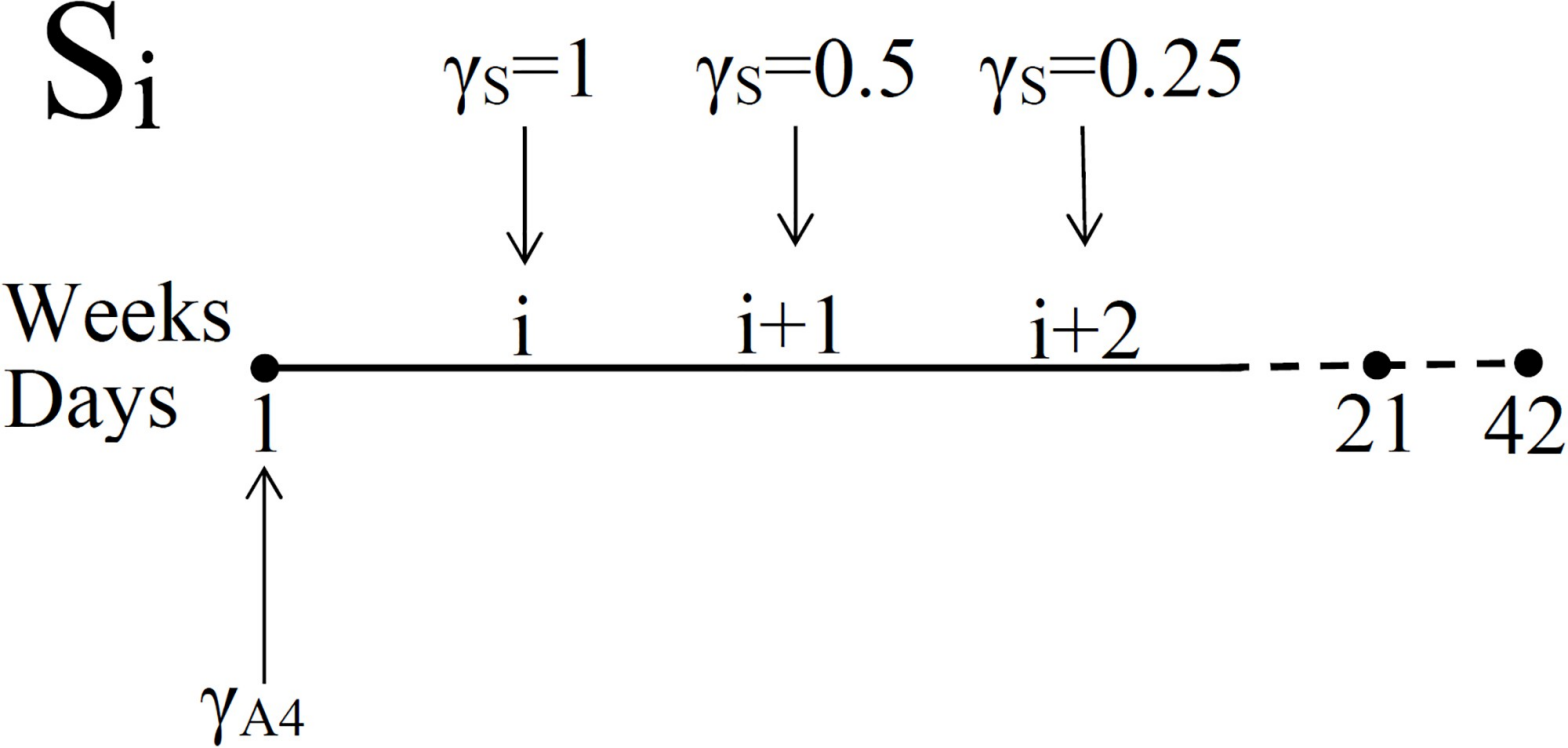
Schedules for administering steroid with doses *γ*_*S*_ = 1, 0.5 and 0.25 mg/kg⋅d. $\gamma_{A_{4}}$ is a dose of anti-CTLA-4, is administered at days 1, 21 and 42.

For the mice experiment settings [9], the tumor was treated with steroids from day 7 to the end-day, *t* = 23. So we may therefore take *f*_*S*_(*t*) = 0 if *t* < 7 and = 1 if *t* ≥ 7. *A*_4_ was injected in days 13, 16 and 19. Fig 5 shows the level of T cells at the end-time under *A*_4_ alone, *S* alone, and under the combination *A*_4_ + *γ*_*S*_. We see that under *A*_4_, the IL-12 level is clearly above the control case, under *A*_4_ + *γ*_*S*_ it decreases below the control case, and the level under *S* alone is extremely small. This pattern is in agreement with [9] Fig 6a and 6b (for convenience we have added these figures in the S1 Appendix).

### Clinical trials in silico

Having established qualitative agreement with some experimental results, we proceed to use the model in clinical trials framework, where treatments according to [71, 72] proceed in 3-week cycles with ipilimumab administered (by infusion) on the first day of each of the first three cycles, at dose 3 mg/kg⋅d.

The average weight of people in the United States is 82 kg, which corresponds approximately to volume of 82 × 10^3^ cm^3^, this means that $\gamma_{A_{4}}=3\times 10^{-6}$ g/cm^3^.

We shall simulate clinical trials seven cycles, so that

$$\begin{array}{c} f_{A_{4}}\left(t\right)=\{\begin{array}{cc} e^{-\mu_{A_{4}}t}, & \text{for}\;0\leq t<21,\\ e^{-\mu_{A_{4}}t}+e^{-\mu_{A_{4}}\left(t-21\right)}, & \text{for}\;21\leq t<42,\\ e^{-\mu_{A_{4}}t}+e^{-\mu_{A_{4}}\left(t-21\right)}+e^{-\mu_{A_{4}}\left(t-42\right)}, & \text{for}\;42\leq t\leq 63,\\ 0, & \text{for}\;t>63 \end{array} \end{array}$$

where $\mu_{A_{4}}$ is the half-life of *A*_4_.

The half-life of prednisone is 3–4 hours [73]; we take the average 3.5 hours, or equivalently, 0.15 days. Hence, *μ*_*S*_ = ln 2/0.15 = 4.62 d^−1^ and we may therefore assume that prednisone administered in one day has negligible level in the following days. On the other hand, since prednisone is usually taken in pills several times a day, we may take the level of the dose to remain constant during the day. Hence we define *f*_*S*_(*t*) in Eq (16) as follows:

$$\begin{array}{c} f_{S}\left(t\right)=\{\begin{array}{cc} \text{1}, & \text{during}\;\text{the}\;\text{week}\;\text{that}\;\text{prednisone}\;\text{is}\;\text{administered},\\ 0, & \text{otherwise}. \end{array} \end{array}$$

According to Aldea et al. [74], for non-severe irAEs, corticosteroid is given at daily amount 0.2 to 0.4 mg/kg for 2 to 3 weeks and then gradually tapered off during the following 2 to 4 weeks; but in the case of significant risk of severe irAEs, it is given in the same schedule in larger amount of 0.7 to 1 mg/kg per day.

We consider the following schedules for administering prednisone (represented schematically in Fig 6): Each treatment is 70 days long with

$$\begin{array}{ccc} S_{i}:\;\gamma_{S} & =\text{1}\;\text{mg/kg}\cdot \text{d}\;\text{in}\;\text{week}\;i\\ & =\text{0.5}\;\text{mg/kg}\cdot \text{d}\;\text{in}\;\text{week}\;i+1\\ & =\text{0.25}\;\text{mg/kg}\cdot \text{d}\;\text{in}\;\text{week}\;i+2\\ & =\text{0}\;\text{in}\;\text{all}\;\text{other}\;\text{weeks}, & \text{where}\;i=1,\ldots ,7\text{.} \end{array}$$ (23)

Note that 1 mg/kg⋅d, 0.5 mg/kg⋅d and 0.25 mg/kg⋅d correspond to 10^−6^, 0.5 × 10^−6^ and 0.25 × 10^−6^ in g/cm^3^⋅d, respectively.

In what follows we use a simplified notation where *T*_*α*_(*t*) denotes the average concentration of TNF-*α* in the tumor at time *t*, and *T*_*α*,con_(*t*) denotes *T*_*α*_(*t*) in the control case (no drugs). Since hypophysitis does not occur in the control case, we assume that toxicity associated with this irAE is represented by the excess of *T*_*α*_(*t*) over *T*_*α*,con_(*t*), and take the average of [*T*_*α*_(*t*) − *T*_*α*,con_(*t*)]^+^ over 70 days to represent the risk of hypophysitis; we denote this average by *T*_*α*,ave_(70), and denote the tumor volume at time *t* by *V*(*t*).

**Fig 7:**
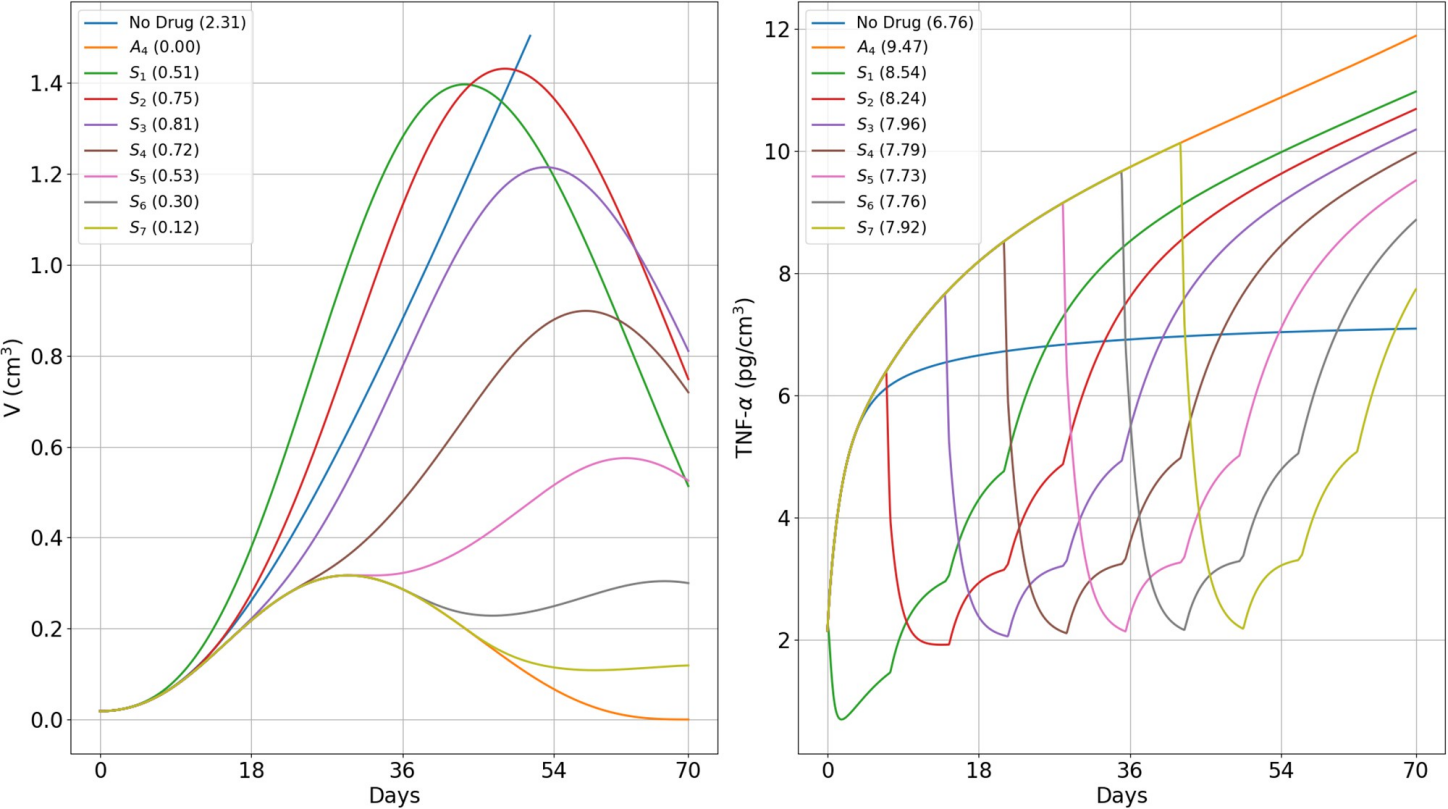
Different protocols of treatment with prednisone and anti-CTLA-4. (*S*_1_) − (*S*_7_): Tumor volume (left) after 10 weeks and average TNF-*α* (right) *A*_4_ is injected in the first day of each cycle, at $\gamma_{A_{4}}=3\times 10^{-6}$ g/cm^3^⋅d. *S* is given at various schedules (*S*_1_)–(*S*_7_) as in (23) at *γ*_*S*_ = 10^−6^ g/cm^3^⋅d (1 mg/kg⋅d), *γ*_*S*_ = 0.5 × 10^−6^ g/cm^3^⋅d (0.5 mg/kg⋅d), and *γ*_*S*_ = 0.25 × 10^−6^ g/cm^3^⋅d (0.25 mg/kg⋅d). (a) The 10-week end-time tumor volumes are displayed on the left panels and the average of TNF-*α* taken over the levels that exceed the no-drug case, *T*_*α*,ave_, on the right panel. Tumor volume at *t* = 0 is 0.01 cm^3^.

Fig 7 left panel shows the profile of *T*_*α*_(*t*) for each of the schedules *S*_*i*_; the column indicates the corresponding volume *V*(70), or *V*(70;*S*_*i*_). Fig 7 right panel shows the profile of *T*_*α*_(*t*) for each schedule, and the column indicates the corresponding value of *T*_*α*,ave_(70), or *T*_*α*,ave_(70; *S*_*i*_); *V*(70, *A*_4_) and *T*_*α*,ave_(70, *A*_4_) correspond to the case when no steroids are given. We want to determine by the end of the first 3 cycles (day 70) in which anti-CTLA-4 is administered, when is the optimal time to start treatment with prednisone in order to keep toxicity as small as possible, while still decreasing tumor growth, and hopefully also keep volume as small as possible by the end-time of prednisone treatment, which we take to be day 126.

From Fig 7 we see that as *i* increases from 1 to 3, *V*(70, *S*_*i*_) increases and *T*_*α*,ave_(70, *S*_*i*_) decreases, and as *i* increases from 5 to 7, *V*(70, *S*_*i*_) decreases and *T*_*α*,ave_(70, *S*_*i*_) increases. So, there is a trade-off between tumor volume and associated toxicity. Interestingly, in the intermediate cases of *i* = 4 and *i* = 5,

$$\begin{array}{c} V\left(70,S_{5}\right)<V\left(70,S_{4}\right)\;\text{and}\;T_{\alpha ,\text{ave}}\left(70,S_{5}\right)<T_{\alpha ,\text{ave}}\left(70,S_{4}\right), \end{array}$$

which mean that schedule *S*_5_ is better than schedule *S*_4_.

Fig 7 right panel shows that *T*_*α*,ave_(70, *A*_4_) = 9.47 pg/cm^3^. Although it is not possible to associate quantitatively the risk of hypophysitis (which is 10% for patients of NSCLC and metastatic melanoma) for each *S*_*i*_, it is clear that *S*_1_ and *S*_2_ schedules pose the largest risk (with *T*_*α*,*ave*_(70)) of above 8.24 pg/cm^3^, and even *S*_3_ and *S*_7_ have considerable risk. This means that steroid treatment should not start too early and not very late. The above comparison between *S*_4_ and *S*_5_ suggests that *S*_5_ and possibly *S*_6_, are the optimal schedules.

**Fig 8:**
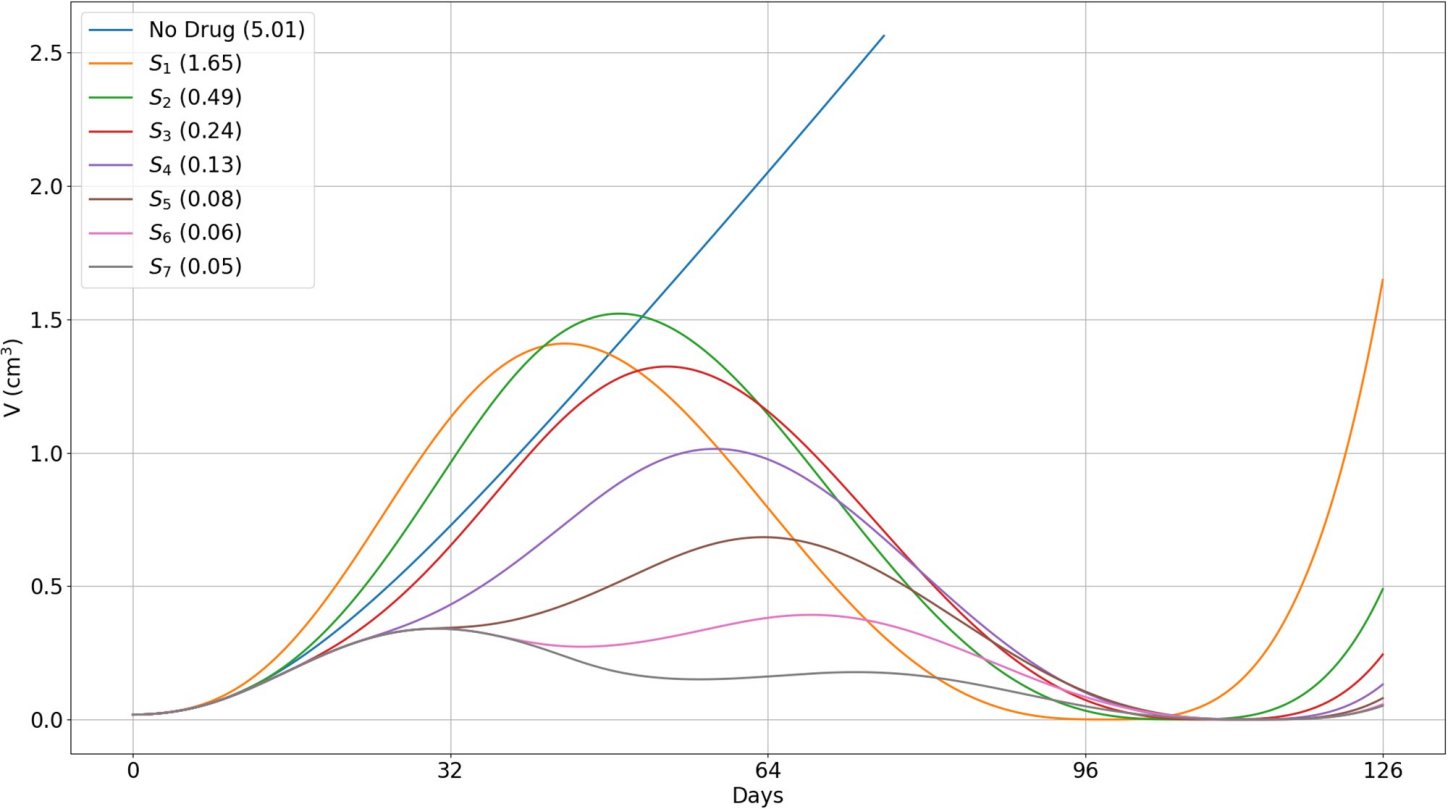
Different protocols of treatment with prednisone and anti-CTLA-4. (*S*_1_) − (*S*_7_): Tumor volume. *A*_4_ is injected in the first day of each cycle, at $\gamma_{A_{4}}=3\times 10^{-6}$ g/cm^3^⋅d. *S* is given at various schedules (*S*_1_)–(*S*_7_) as in (23) at *γ*_*S*_ = 10^−6^ g/cm^3^⋅d (1 mg/kg⋅d), *γ*_*S*_ = 0.5 × 10^−6^ g/cm^3^⋅d (0.5 mg/kg⋅d), and *γ*_*S*_ = 0.25 × 10^−6^ g/cm^3^⋅d (0.25 mg/kg⋅d). Tumor volume for 18 weeks. Tumor volume at *t* = 0 is 0.01 cm^3^.

In Fig 8 we followed the growth of tumor volume after treatment, until day 126. We see that as *i* increases from 1 to 7, the volume *V*(126, *S*_*i*_) decreases as *i* increases. More specifically, *V*(126, *S*_1_) > 3*V*(126, *S*_2_), *V*(126, *S*_2_) > 2*V*(126, *S*_3_), *V*(126, *S*_3_) is nearly equal to 2*V*(126, *S*_4_), and *V*(126, *S*_4_) is nearly equal to 2*V*(126, *S*_5_). This says, even more strongly than Fig 7, that treatment with prednisone should not begin in the early weeks after treatment with ipilimumab had begun. The volumes *V*(126, *S*_*i*_) for *i* = 5, 6, 7, are very close to each other, but since *S*_7_ incurs significant higher toxicity, options *S*_5_ and *S*_6_ still appear to the best.

**Fig 9:**
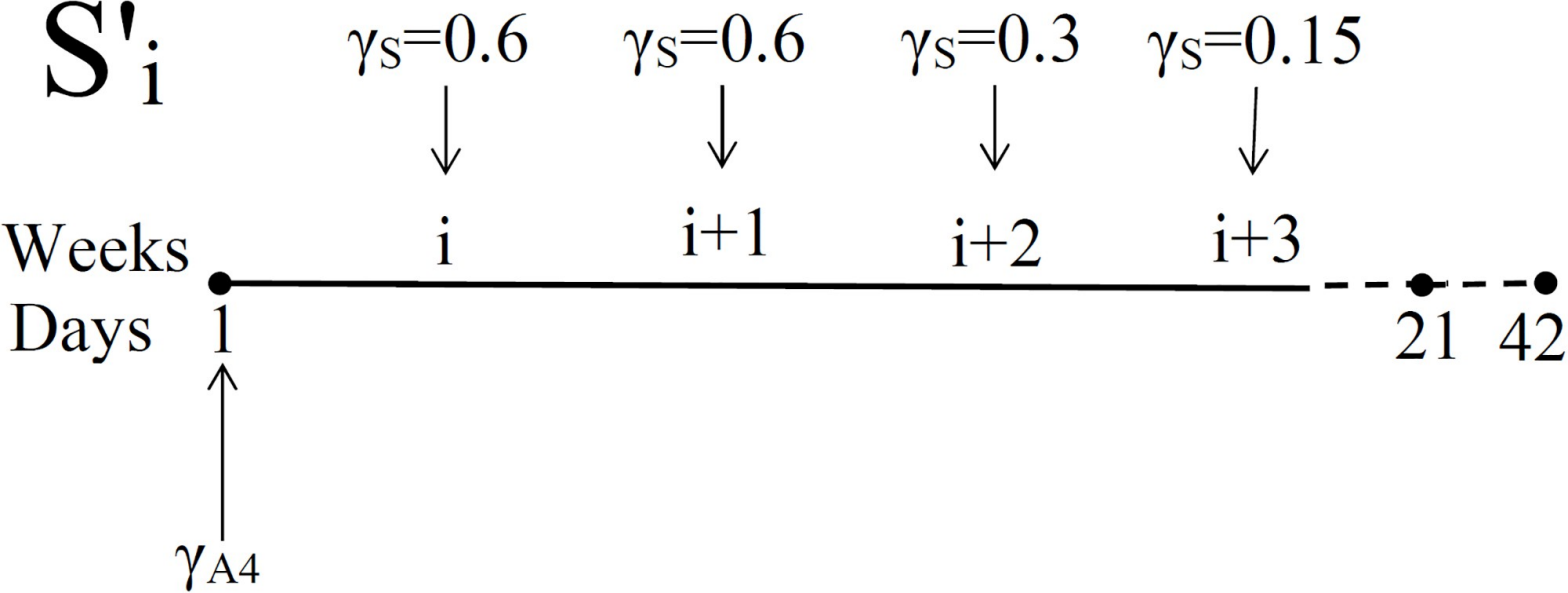
Schedules for administering steroid with doses *γ*_*S*_ = 0.6, 0.6, 0.3 and 0.15 mg/kg⋅d. $\gamma_{A_{4}}$ is the dose of anti-CTLA-4, administered at days 1, 21 and 42.

We may use the model to consider other protocols for prednisone administration. For example, the one shown in Fig 9, where

$$\begin{array}{ccc} S_{i}^{\prime }:\;\gamma_{S} & =\text{0.6}\;\text{mg/kg}\cdot \text{d}\;\text{in}\;\text{weeks}\;i\;\text{and}\;i+1\\ & =\text{0.3}\;\text{mg/kg}\cdot \text{d}\;\text{in}\;\text{week}\;i+2\\ & =\text{0.15}\;\text{mg/kg}\cdot \text{d}\;\text{in}\;\text{week}\;i+3\\ & =\text{0}\;\text{in}\;\text{the}\;\text{remaining}\;\text{weeks}\;\text{of}\;\text{the}\;\text{cycle}, & \text{where}\;i=1,\ldots ,7\text{.} \end{array}$$ (24)

**Fig 10:**
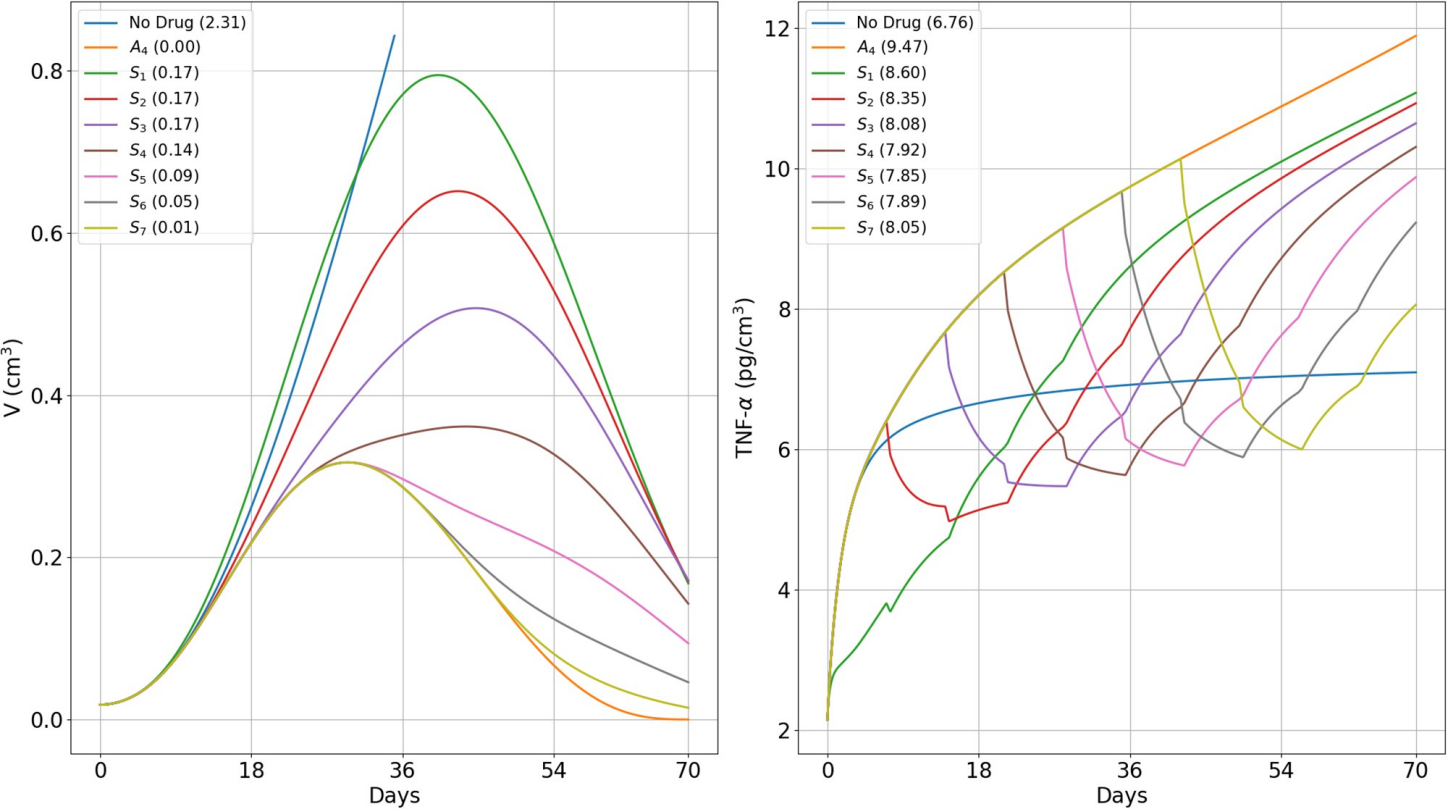
Different protocols of treatment with prednisone and anti-CTLA-4. $\left(S_{1}^{\prime }\right)-\left(S_{7}^{\prime }\right)$ : Tumor volume (left) after 10 weeks and average TNF-*α* (right). *A*_4_ is injected in the first day of each cycle, at $\gamma_{A_{4}}=3\times 10^{-6}$ g/cm^3^⋅d. *S* is given at various schedules ($S_{1}^{\prime }$)–($S_{7}^{\prime }$) as in (23) at *γ*_*S*_ = 0.6 × 10^−6^ g/cm^3^⋅d (0.6 mg/kg⋅d), *γ*_*S*_ = 0.3 × 10^−6^ g/cm^3^⋅d (0.3 mg/kg⋅d), and *γ*_*S*_ = 0.15 × 10^−6^ g/cm^3^⋅d (0.15 mg/kg⋅d). The 10-week end-time tumor volumes are displayed on the left panels and the average of TNF-*α* taken over the levels that exceed the no-drug case, *T*_*α*,ave_, on the right panel. Tumor volume at *t* = 0 is 0.01 cm^3^.

**Fig 11:**
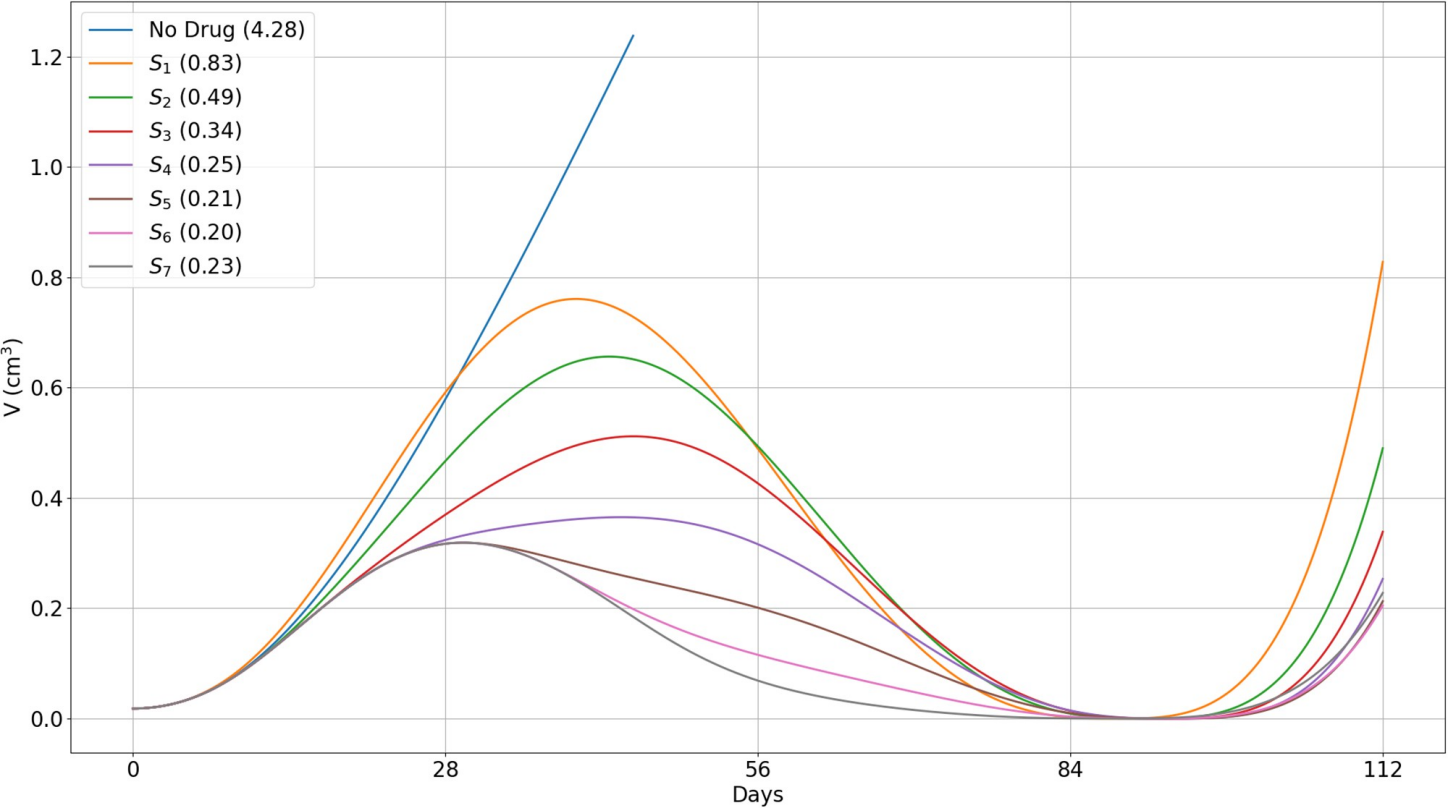
Different protocols of treatment with prednisone and anti-CTLA-4. $\left(S_{1}^{\prime }\right)-\left(S_{7}^{\prime }\right)$ : Tumor volume. *A*_4_ is injected in the first day of each cycle, at $\gamma_{A_{4}}=3\times 10^{-6}$ g/cm^3^⋅d. *S* is given at various schedules ($S_{1}^{\prime }$)–($S_{7}^{\prime }$) as in (23) at *γ*_*S*_ = 0.6 × 10^−6^ g/cm^3^⋅d (0.6 mg/kg⋅d), *γ*_*S*_ = 0.3 × 10^−6^ g/cm^3^⋅d (0.3 mg/kg⋅d), and *γ*_*S*_ = 0.15 × 10^−6^ g/cm^3^⋅d (0.15 mg/kg⋅d). Tumor volume for 16 weeks. Tumor volume at *t* = 0 is 0.01 cm^3^.

Figs 10 and 11 show simulations similar to those in Figs 7 and 8, and we again conclude that treatment with prednisone should start not too early and not very late; as before, intermediate options *S*_5_, and possibly *S*_6_, are optimal.

## 4 Conclusion

Immune checkpoint inhibitors (ICI) have been introduced in recent years in the treatment of NSCLC and metastatic melanoma. However, by inhibiting negative regulation of inflammatory T cells, this treatment elicits toxicity which results in severe adverse events, including damage to organs such as the pituitary gland, where 10% of patients receiving CTLA-4 inhibitor (ipilimumab) develop hypophysitis. Steroids are known to decrease the number of inflammatory T cells and may therefore be used with ICI in order to reduce the risk of adverse events. But steroids also have the effect of increasing the tumor. The opposite effects of steroid on patient’s health raises the following question: What is the optimal time for steroid initiation. This question was considered in several studies. Meta-analysis of such studies shows that patients whose treatment with steroid began less than 2 months from ICI initiation had shorter OS than those who began treatment after 2 months [13].

In this paper we consider patients of NSCLC or metastatic melanoma who are treated with CTLA-4 inhibitor (ipilimumab), and thus are at risk of severe adverse events, such as permanent damage to a vital organ. We take the pituitary gland to represent these organs, since hypophysitis occurs at significantly larger percentage than other damaged organs.

We assume that the risk of hypophysitis is the result of toxicity due to treatment with CTLA-4 inhibitor, and we represent the level of toxicity by the concentration of TNF-*α* in the tumor. We developed a mathematical model in order to determine optimal scheduling of treatment with steroid. The model is represented by a dynamical system of partial differential equations with variables that include cancer cells, immune cells, cytokines, and the two drug drugs (ipilimumab and prednisone). Most of the equations, as well as the estimates of the parameters and their sensitivity analysis, are given in Supplement Information; we have shown that the model simulations agree with mice experiments in [9, 74].

We then proceeded to simulate clinical trials in three 3-week cycles: Ipilimumab is infused at the beginning of each of the first three cycles, and steroid, with schedule *S*_*i*_, begins at week *i*, for 3 or 4 weeks duration, where a large dose given in the early week(s) is tapered off during the remaining weeks. We took *i* = 1, 2, …, 7, and treatment was evaluated at day 70.

We simulated the outcome of each schedule up to day 126, and computed two quantities:

$$\begin{array}{cc} T_{\alpha ,\text{ave}}\left(70,S_{i}\right) & =\text{Average}\;\text{of}\;T_{\alpha }-T_{\alpha ,\text{con}}\;\text{at}\;\text{day}\;\text{70},\\ V\left(70,S_{i}\right),V\left(126,S_{i}\right) & =\text{Volume}\;\text{of}\;\text{tumor}\;\text{at}\;\text{days}\;\text{70,}\;\text{126}, \end{array}$$

where *T*_*α*,con_ is the average concentration of TNF-*α* in the control case (no drugs) when we know that hypophysitis does not occur; *T*_*α*,con_(*t*) is approximately 6.76 pg/cm^3^. In the case when no steroid is given, *T*_*α*,ave_(70) = 9.47 pg/cm^3^, and this level of toxicity corresponds to 10% risk of hypophysitis. This gives us some idea about the risk of hypophysitis in terms of the level of *T*_*α*,ave_(70, *S*_*i*_).

The values of the pairs (*T*_*α*_(70, *S*_*i*_), *V*(70, *S*_*i*_)) can be used to compare the benefits of each schedule of steroid administration and the optimal schedules were supported by the values of *V*(126, *S*_*i*_). In particular, we conclude that treatment with prednisone should not start in the first 4 weeks (too early) and not in week 7 (very late); schedules *S*_5_, and possibly *S*_6_, are the optimal ones; more generally, treatment with prednisone should start as soon as tumor volume, under the effect of CTLA-4 inhibitor alone, begins to decrease. We can also use the same pairs to compare the benefits of short time administration of steroid at total high dose with longer time administration at lower doses. For example, in, 3-week treatment with doses of 1, 0.5 and 0.25 mg/kg⋅d, we get, by Figs 7 and 8,

$$\begin{array}{c} T_{\alpha }\left(70,S_{5}\right)=7.73\;\text{pg/cm}{}^{3},\;V\left(70,S_{5}\right)=0.53\;\text{cm}{}^{3},\;V\left(126,S_{5}\right)=0.08\;\text{cm}{}^{3}, \end{array}$$

while in, 4-week treatment with 0.6, 0.6, 0.3 and 0.15 mg/kg⋅d, we get, by Figs 10 and 11,

$$\begin{array}{c} T_{\alpha }\left(70,S_{5}^{\prime }\right)=7.85\;\text{pg/cm}{}^{3},\;V\left(70,S_{5}^{\prime }\right)=0.09\;\text{cm}{}^{3},\;V\left(112,S_{5}^{\prime }\right)=0.21\;\text{cm}{}^{3}, \end{array}$$

i.e., slightly more toxicity and larger cancer volume than in the 3-week treatment (already by day 112) with higher doses. Hence the particular 3-week schedule of steroid treatment is better than the 4-week schedule.

The paper has several limitations:

1. The conclusion arrived at on optimal scheduling of prednisone were based on short term evaluation. In order to gain more confidence in such conclusions, long term evaluations will be needed whereby anti-CTLA-4 is administered for larger number of cycles, and assessment is done at some times during treatment and, longer times, after the end of treatment.
2. The strategy for administering prednisone used in this paper is to start with a high dose, and continue decreasing the level of doses in the following weeks. But many other strategies could be considered, for example, intermittent treatment with high/medium dose of prednisone on weeks 2, 4 and 6, or 3, 5 and 6.
3. Toxicity was represented only by TNF-*α* that is produced by *T*_1_ cells; IL-1 and IL-6 were omitted since they are secreted primarily by macrophages [36, 37] and, hence, are not appreciably affected by CTLA-4 inhibitor. If we were to include macrophages with its associated cytokines, the model’s complexity will be significantly increased and so will the level of inaccuracies in estimating parameters.
4. The mechanism that leads from toxicity to hypophysitis was not considered, and the level of toxicity, by TNF-*α*, was taken only within the tumor.
5. Clinical studies shows that 10% of NSCLC and metastatic melanoma patients treated with CTLA-4 inhibitor develop hypophysitis, and our model shows that the corresponding level of TNF-*α* about the control level (*T*_*α*,ave_(70)) is 9.47 pg/ml. However, we are unable to fit a lower threshold of TNF-*α* with exact percentage of patients that will develop hypophysitis.
6. The level of TNF-*α* in homeostasis varies among people, and the average level among various studies varies greatly.

Nevertheless, the mathematical model provides a conceptual framework for assessing options of administering steroids with ICI, and could serve a useful prognostic tool in designing clinical trials with CTLA-4 inhibitor in combination with PD-1 or PD-L1 inhibitor, and chemotherapeutic drugs.

## Supporting information

S1 File Model equations, Parameters estimates, sensitivity analysis, numerical methods and tables of parameters. (PDF)

S1 Appendix (PDF)
