# Supplementary material for: Optimal timing of steroid initiation in response to CTLA-4 antibody in metastatic cancer: A mathematical model
Source: PLoS One. 2022 Nov 10;17(11):e0277248. doi: 10.1371/journal.pone.0277248 (PMC9648769; doi:10.1371/journal.pone.0277248)
Supplement: S1 File — (PDF) [file pone.0277248.s001.pdf]

Optimal timing of steroid initiation in response to CTLA-4 antibody in metastatic cancer: A mathematical model Supplement Information

Nourridine Siewe[1*], Avner Friedman[2],

1 School of Mathematical Sciences, College of Science, Rochester Institute of Technology, Rochester, New York, U.S.A.
2Department of Mathematics, The Ohio State University, Columbus, Ohio, U.S.A.

* nourridine@aims.ac.za

1 Model Model

We assume that the combined density of all cells in the tumor region $\Omega(t)$ remains constant in space and time,

$$D + T_1 + T_8 + T_r + C = \text{constant} = \theta. \tag{1}$$

1.1 Equation for D

Immature dendritic cells (D_0) are activated by HMGB-1 produced by necrotic cancer cells [1–3]. We represent this activation by a Michaelis-Menten rate $\lambda_{DC} D_0 C/(K_C + C)$, where D_0 is the source of immature dendritic cells. Hence, the dynamics of DCs is given by

$$\frac{\partial D}{\partial t} + \nabla \cdot (\mathbf{u} D) - \delta_D \nabla^2 D = \lambda_{DC} D_0 \frac{C}{K_C + C} - \mu_D D, \tag{2}$$

where δ_D is the diffusion coefficient and μ_D is the death rate.

1.2 Equations for T_1 and T_8

Naive CD4$^+$ T cells, T_{10}, differentiate into T_1 cells under I_{12} [4] and T_α [5] environment, a process inhibited by T_r [6]. The proliferation of activated T_1 cells is enhanced by I_2 [7,8]. Both processes of activation and proliferation are inhibited by the complex B7/CTLA-4 (Q), by a factor $1/(1 + Q/K_{TQ})$, and prednisone (S) depletes T_1 cells [9]. Hence, T_1 satisfies the following equation:

$$\begin{aligned} \frac{\partial T_1}{\partial t} &+ \nabla \cdot (\mathbf{u} T_1) - \delta_{T_1} \nabla^2 T_1 = \\ &\left[T_{10} \left(\lambda_{T_1 I_{12}} \frac{I_{12}}{K_{I_{12}} + I_{12}} + \lambda_{T_1 T_\alpha} \frac{T_\alpha}{K_{T_\alpha} + T_\alpha} \right) \frac{1}{1 + T_r/K_{TT_r}} + \lambda_{T_1 I_2} T_1 \frac{I_2}{K_{I_2} + I_2} \right] \\ &\times \frac{1}{1 + Q/K_{TQ}} - \mu_{T_1} T_1 - \mu_{ST} S T_1. \end{aligned} \tag{3}$$

Similarly,

$$\frac{\partial T_8}{\partial t} + \nabla \cdot (\mathbf{u} T_8) - \delta_{T_8} \nabla^2 T_8 = \tag{4}$$
$$\left[T_{80}\left(\lambda_{T_8 I_{12}} \frac{I_{12}}{K_{I_{12}} + I_{12}} + \lambda_{T_8 T_\alpha} \frac{T_\alpha}{K_{T_\alpha} + T_\alpha}\right) \frac{1}{1 + T_r/K_{TT_r}} + \lambda_{T_8 I_2} T_8 \frac{I_2}{K_{I_2} + I_2}\right]$$
$$\times \frac{1}{1 + Q/K_{TQ}} - \mu_{T_8} T_8 - \mu_{ST} S T_8;$$

note that T_r also controls the activation of T_8 cells [10].

1.3 Equation for T_r

The activation of T_r is induced by TGF-β [11], so that

$$\frac{\partial T_r}{\partial t} + \nabla \cdot (\mathbf{u} T_r) - \delta_{T_r} \nabla^2 T_r = \lambda_{T_r T_\beta} T_{10} \frac{T_\beta}{K_{T_\beta} + T_\beta} - \mu_{T_r} T_r. \tag{5}$$

1.4 Equation for C

We assume logistic growth of cancer cells, with carrying capacity C_M, to account for their competition for space and nutrients. Cancer cells are killed primarily by CD8$^+$ T cells, hence

$$\frac{\partial C}{\partial t} + \nabla \cdot (\mathbf{u} C) - \delta_C \nabla^2 C = \lambda_C C \left(1 - \frac{C}{C_M}\right) - \mu_{T_8 C} T_8 C - \mu_C C. \tag{6}$$

1.5 Equations for cytokines

IL-2 is produced by T_1 cells [7, 8], so that

$$\frac{\partial I_2}{\partial t} - \delta_{I_2} \nabla^2 I_2 = \lambda_{I_2 T_1} T_1 - \mu_{I_2} I_2, \tag{7}$$

where μ_{I_2} is a degradation rate. Note that the diffusion coefficient of cytokines is several orders of magnitude larger than the diffusion coefficient of cells, hence their advection velocity is negligible relative to their diffusion, and may therefore be dropped.

IL-12 is secreted by DCs [12, 13]. IL-12 is also secreted by iosinophils as a result of toxicity due to immunotherapy [14], which we assume to be proportional to A_4. Activated Th1 cells are the main receptors of IL-12, which means that they decrease IL-12 ligands in the process of being activated [15]. Hence,

$$\frac{\partial I_{12}}{\partial t} - \delta_{I_{12}} \nabla^2 I_{12} = \lambda_{I_{12} D} D + \lambda_{I_{12} A_4} A_4 - \mu_{I_{12} T_1} T_1 \frac{I_{12}}{K_{I_{12}} + I_{12}} - \mu_{I_{12}} I_{12}, \tag{8}$$

where $\mu_{I_{12}}$ is a degradation rate.

TNF-α is produced by T_1 cells [5], so that

$$\frac{\partial T_\alpha}{\partial t} - \delta_{T_\alpha} \nabla^2 T_\alpha = \lambda_{T_\alpha T_1} T_1 - \mu_{T_\alpha} T_\alpha. \tag{9}$$

TGF-β is produced by cancer cells [16] and T_r cells [6], hence

$$\frac{\partial T_\beta}{\partial t} - \delta_{T_\beta} \nabla^2 T_\beta = \lambda_{T_\beta C} C + \lambda_{T_\beta T_r} T_r - \mu_{T_\beta} T_\beta. \tag{10}$$

1.6 Equation for B7 (B_7), CTLA-4 (P_A) and B7/CTLA-4 (Q)

CTLA-4 is a receptor expressed on activated T_1 and T_8 cells [17] and the complex B7/CTLA-4 blocks the activities of these cells [17, 18]. CTLA-4 is constitutively expressed on T_r cells, but its activity is not blocked by the complex B7/CTLA-4 [19]. We assume that the number of CTLA-4 proteins per cell is the same for T_1 and T_8 cells, but different for T_r cells, by a factor κ_T. We denote by ρ_{P_A} the ratio between the mass of all CTLA-4 proteins in one T cell to the mass of this cell, so that

$$P_A = \rho_{P_A}(T_1 + T_8 + \kappa_T T_r).$$

The coefficient ρ_{P_A} is constant when no anti-CTLA-4 drug is administered. In this case, to a change in T (T_1, T_8, T_r), given by $\partial T/\partial t$, there corresponds a change of P_A, given by $\rho_{P_A}\partial T/\partial t$. Similar changes in P_A arises from the terms of diffusion and advection, so that

$$\begin{aligned}
&\frac{\partial P_A}{\partial t} + \nabla\cdot(\mathbf{u}P_A) - \delta_T\nabla^2 P_A = \\
&\quad \rho_{P_A}\Bigg\{ \Bigg[\Bigg((\lambda_{T_1I_{12}}T_{10} + \lambda_{T_8I_{12}}T_{80})\frac{I_{12}}{K_{I_{12}}+I_{12}} + (\lambda_{T_1T_\alpha}T_{10} + \lambda_{T_8T_\alpha}T_{80})\frac{T_\alpha}{K_{T_\alpha}+T_\alpha}\Bigg) \\
&\quad \times \frac{1}{1+T_r/K_{TT_r}} + (\lambda_{T_1I_2}T_1 + \lambda_{T_8I_2}T_8)\frac{I_2}{K_{I_2}+I_2}\Bigg]\frac{1}{1+Q/K_{TQ}} \\
&\quad + \kappa_T\ \lambda_{T_rT_\beta}T_{10}\frac{T_\beta}{K_{T_\beta}+T_\beta} - (\mu_{T_1}T_1 + \mu_{T_8}T_8 + \mu_{ST}S(T_1+T_8) + \kappa_T\mu_{T_r}T_r)\Bigg\}.
\end{aligned}$$

When anti-CTLA-4 drug (A_4) is applied, CTLA-4 is depleted at a rate proportional to A_4, and, in this case, the ratio $P_A/(T_1 + T_8 + \kappa_T T_r)$ may change. In order to include in the model both cases, with and without anti-CTLA-4, we replace ρ_{P_A} in the above equation by $P_A/(T_1 + T_8 + \kappa_T T_r)$. Hence,

$$\begin{aligned}
&\frac{\partial P_A}{\partial t} + \nabla\cdot(\mathbf{u}P_A) - \delta_T\nabla^2 P_A = \frac{P_A}{(T_1+T_8+\kappa_T T_r)} \\
&\quad \times\Bigg\{ \Bigg[\Bigg((\lambda_{T_1I_{12}}T_{10} + \lambda_{T_8I_{12}}T_{80})\frac{I_{12}}{K_{I_{12}}+I_{12}} + (\lambda_{T_1T_\alpha}T_{10} + \lambda_{T_8T_\alpha}T_{80})\frac{T_\alpha}{K_{T_\alpha}+T_\alpha}\Bigg) \\
&\quad \times \frac{1}{1+T_r/K_{TT_r}} + (\lambda_{T_1I_2}T_1 + \lambda_{T_8I_2}T_8)\frac{I_2}{K_{I_2}+I_2}\Bigg]\frac{1}{1+Q/K_{TQ}} \qquad (11) \\
&\quad + \kappa_T\ \lambda_{T_rT_\beta}T_{10}\frac{T_\beta}{K_{T_\beta}+T_\beta} - (\mu_{T_1}T_1 + \mu_{T_8}T_8 + \mu_{ST}S(T_1+T_8) + \kappa_T\mu_{T_r}T_r)\Bigg\} \\
&\quad - \mu_{P_AA_4}P_AA_4,
\end{aligned}$$

where $\mu_{P_AA_4}$ is the depletion rate of CTLA-4 by anti-CTLA-4.

Ligand B7 is expressed on dendritic cells, so that

$$B_7 = \rho_{B_7}D, \quad \rho_{B_7}\text{=constant}.$$

B7 and CTLA-4 from the complex B7/CTLA-4 (Q) with association and disassociation rates $\alpha_{B_7P_A}$ and μ_Q, respectively:

$$B_7 + P_A \underset{\mu_Q}{\overset{\alpha_{B_7P_A}}{\rightleftharpoons}} Q.$$

We assume that the half-life of Q is very short [20, 21], so that we may approximate the dynamics of Q by the steady state, $\alpha_{B_7P_A}B_7P_A = \mu_Q Q$, or

$$Q = \sigma B_7 P_A, \tag{12}$$

where $\sigma = \alpha_{B_7P_A}/\mu_Q$.

1.7 Equations for A_4 and S

The concentration of anti-CTLA-4 satisfies the equation

$$\frac{\partial A_4}{\partial t} - \delta_{A_4}\nabla^2 A_4 = \underbrace{\gamma_{A_4} f_{A_4}(t)}_{\text{source}} - \underbrace{\mu_{P_A A_4} P_A A_4}_{\text{depletion through blocking CTLA-4}} - \underbrace{\mu_{A_4} A_4}_{\text{degradation}} \tag{13}$$

where the drug is injected at dose γ_{A_4} several times during treatment, and its actual strength at time t is $\gamma_{A_4} f_{A_4}(t)$.

Similarly, the concentration of prednisone satisfies the following equation:

$$\frac{\partial S}{\partial t} - \delta_S \nabla^2 S = \underbrace{\gamma_S f_S(t)}_{\text{source}} - \underbrace{\mu_{TS}(T_1 + T_8)S}_{\text{depletion through blocking } T_1 \text{ and } T_8} - \underbrace{\mu_S S}_{\text{degradation}} . \tag{14}$$

1.8 Boundary conditions

We assume that the tumor boundary, $\partial\Omega(t)$, is moving with velocity of the cells, that is

$$V_n = \mathbf{u} \cdot \mathbf{n} \tag{15}$$

where $\mathbf{n}$ is the outward normal at boundary and V_n is the velocity of the free boundary of the tumor in the direction $\mathbf{n}$.

We assume that the inactive CD4$^+$ and CD8$^+$ T cells that migrated from the lymph nodes into the tumor microenvironment have constant densities $\hat{T}_1$ and $\hat{T}_8$, respectively, at the tumor boundary, and that they are activated by IL-12 upon entering the tumor. We then have the following conditions at the tumor boundary:

$$\begin{aligned} \frac{\partial T_1}{\partial t} &+ \sigma_0 \frac{I_{12}}{K_{I_{12}} + I_{12}} (T_1 - \hat{T}_1)^+ = 0, \\ \frac{\partial T_8}{\partial r} &+ \sigma_0 \frac{I_{12}}{K_{I_{12}} + I_{12}} (T_8 - \hat{T}_8)^+ = 0 \quad \text{at } r = R(t). \end{aligned} \tag{16}$$

We impose no-flux boundary condition on all the remaining variables:

$$\text{No flux for } C,\ T_r,\ D,\ I_2,\ I_{12},\ T_\alpha,\ T_\beta,\ P_A,\ A_4, \text{ and } S \text{ at } r = R(t); \tag{17}$$

it is tacitly assumed here that CTLA-4 become actives only after the T cells are already inside the tumor.

We prescribe initial conditions, in units of g/cm^3,

$$\begin{aligned} &C = 0.41,\ T_1 = 10^{-3},\ T_8 = 2.4 \times 10^{-4},\ T_r = 10^{-5},\ D = 10^{-6}, \\ &I_2 = 1.5 \times 10^{-11},\ I_{12} = 5 \times 10^{-11},\ T_\alpha = 4.4 \times 10^{-12},\ T_\beta = 7.4 \times 10^{-9}, \text{ and } R = 0.13 \text{ cm}. \end{aligned} \tag{18}$$

2 Numerical Simulations

All the computations were done using Python 3.5.4. The parameter values of the model equations, except μ_{TS}, μ_{ST}, $\mu_{P_A A_4}$ and C_M, are estimated in Section 3 and are listed in Tables 1 and 2. Parameter sensitivity analysis was performed in Section 4, and the numerical scheme used in the simulations is described in Section 5.

The simulations are carried out in the case of radially symmetric tumor, where $\Omega(t) = \{0 \le r \le R(t)\}$, and radially symmetric variables, that is, functions of (r, t), where $r = |x|$ is the distance of a point x to the origin, and $\mathbf{u} = u\mathbf{e}_r$ where $u = u(r, t)$ and $\mathbf{e}_r$ is the unit vector $x/|x|$. Then Eq. (15) becomes

$$\frac{dR(t)}{dt} = u(R(t), t). \tag{19}$$

From Eq. (1) we then deduce that

$$\frac{dR(t)}{dt} = \frac{\theta}{R^2(t)} \int_0^{R(t)} \left[\sum_{j=2}^{6} \text{R.H.S. of Eqs. (2.j)} \right] r^2 dr \tag{20}$$

3 Parameters Estimates

3.1 Half-saturation

We denote by Z^0 the average density/concentration of species Z. In an expression of the form $Y\dfrac{X}{K_X + X}$ where Y is activated by X, the parameter K_X is called the half-saturation of X. We assume that

$$\frac{X^0}{K_X + X^0}$$

to be not too close to 0 or to 1, and, for simplicity, take it to be 1/2, so that

$$K_X = X^0. \tag{21}$$

3.2 Estimate for T_8^0

Lisiero et al. [22] measured the level of CD8$^+$ T cells in a melanoma model in mice, under IL-12 priming. They reported levels of $7 - 10 \times 10^5$ cells/cm^3 after three days. Since the mass of one cell is approximately 5×10^{-10} g, we take

$$T_8^0 = K_{T_8} = 10^6 \times 5 \times 10^{-10} = 5 \times 10^{-4} \text{ g/cm}^3.$$

3.3 Estimate for T_r^0

The proportion of T_r in prostate cancer was measured in [23] as $T_r = 4\%(T_1 + T_8 + T_r)$. We assume that the proportion of T_r to T cells is the same in melanoma, with $T_1 = 2 \times 10^{-3}$ g/cm^3 [24] and $T_8 = 5 \times 10^{-4}$ g/cm^3. Hence

$$T_r^0 = K_{T_r} = 1.04 \times 10^{-4} \text{ g/cm}^3.$$

We also take

$$K_{TT_r} = 1.04 \times 10^{-4} \text{ g/cm}^3.$$

3.4 Estimate for K_D and D_0

We take the estimate of $D^0 = K_D$ and D_0 from [24], as follows:

$$D^0 = K_D = 4 \times 10^{-4} \text{ g/cm}^3 \text{ and } D_0 = 2 \times 10^{-5} \text{ g/cm}^3.$$

3.5 Estimate for K_{I_2}

The plasma level of IL-2 in advanced stage metastatic melanoma ranges between 4.15–55 pg/ml, with mean 18.85 pg/ml [25]. Hence

$$K_{I_2} = I_2^0 = 1.9 \times 10^{-11} \text{g/cm}^3.$$

3.6 Estimate for $K_{I_{12}}$

Jafarzadeh et al. [26] evaluated the circulating levels of IL-12 in patients with breast cancer, and found that the circulating level of IL-12 in stage 4 was approximately 100 pg/cm^3. We assume that advanced level of melanoma corresponds to stage 4 breast cancer, and take

$$K_{I_{12}} = I_{12}^0 = 10^{-10} \text{g/cm}^3.$$

3.7 Estimate for K_{T_α}

The reported average level of TNF-α in healthy humans varies greatly among studies [27]. We take it to be 4.2 pg/ml [28]; it was higher in [29], and lower in [30]. The serum concentration ratio of TNF-α in NSCLC compared to healthy control was 2 to 1 (42.2 vs. 23 pg/ml) [31]. We take the level of T_α in metastatic cancer to be twice that in health. Hence

$$K_{T_\alpha} = 8.4 \times 10^{-12} \text{ g/cm}^3.$$

3.8 Estimate for K_{T_β}

The average level of TGF-β in primary melanoma tumor line is 36 pg/10^5 cells [32], or 36×10^{-12} g/($10^5 \times 5 \times 10^{-10}$ cm^3), assuming that the volume of 1 cell is 5×10^{-10} g [33]. Hence

$$K_{T_\beta} = T_\beta^0 = 7.2 \times 10^{-9} \text{ g/cm}^3.$$

3.9 Estimate for K_{TQ}

By (12),

$$\frac{1}{1 + Q/K_{TQ}} = \frac{1}{1 + P_A B_7/K'_{TQ}},$$

where $K'_{TQ} = K_{TQ}/\sigma$, and we estimate K'_{TQ}, as in [24], by

$$K'_{TQ} = 4.86 \times 10^{-20} \text{ g}^2/\text{cm}^6.$$

3.10 Estimates for the diffusion coefficients δ_X

Young [34] established the following formula for estimating the diffusion coefficient δ_p of a protein p:

$$\delta_p = \frac{\beta}{M_p^{1/3}}, \tag{22}$$

where M_p is the molecular weight of p and β is a constant. Since for V=VEGF $M_V = 24$ kDa [35] and $\delta_V = 8.64 \times 10^{-2}$ cm^2 d^{-1} [36],

$$\beta = 8.64 \times 10^{-2} \times (24)^{1/3} = 0.25 \text{ cm}^2 \text{ d}^{-1} \text{ (kDa)}^{1/3}.$$

From $M_{I_2} = 16$ kDa [35] and $M_{I_{12}} = 37$ kDa [35], $M_{T_\alpha} = 17.3$ kDa [37], and $M_{T_\beta} = 4.76$ kDa [38], we deduce that $\delta_{I_2} = 9.92 \times 10^{-2}$ cm^2 d^{-1}, $\delta_{I_{12}} = 7.5 \times 10^{-2}$ cm^2 d^{-1}, $\delta_{T_\alpha} = 9.76 \times 10^{-2}$ cm^2 d^{-1}, and $\delta_{T_\beta} = 14.86 \times 10^{-2}$ cm^2 d^{-1}.

We assume that Eq. (22) can be applied also to drugs; since $M_{A_4} = 37$ kDa [39] and $M_S = 360.4$ kDa [40], we get: $\delta_{A_4} = 7.5 \times 10^{-2}$ cm^2 d^{-1}, and $\delta_S = 3.51 \times 10^{-2}$ cm^2 d^{-1}.

3.11 Diffusion coefficients of cells

We take the diffusion coefficient of T cells to be $\delta_T = 8.64 \times 10^{-7}$ cm^2 d^{-1} [24], and assume that all other cell types have, approximately, the same diffusion coefficient, taking

$$\delta_X = 8.64 \times 10^{-7} \text{ cm}^2 \text{ d}^{-1}, \text{ for } X = C,\ D,\ T_1,\ T_8,\ T_r.$$

3.12 Estimate for μ_D

The half-life of activated dendritic cells ranges between 2–5 days [41]. We take the half-life $t^D_{1/2} = 5$ days. Hence,

$$\mu_D = \frac{\ln 2}{5 \text{ d}} = 0.13 \text{ d}^{-1}.$$

3.13 Estimates for μ_{T_1} and μ_{T_8}

The half-life of activated T cells (T_1 and T_8) ranges between 24–76 hours [42]. We take $t^T_{1/2}$ =36 hours. Hence,

$$\mu_{T_1} = \frac{\ln 2}{1.5 \text{ d}} = 0.2 \text{ d}^{-1}, \quad \mu_{T_8} = \frac{\ln 2}{1.5 \text{ d}} = 0.2 \text{ d}^{-1}$$

3.14 Estimate for μ_{T_r}

The half-life of the regulatory T cells is approximately 2.7 days [43]. Hence,

$$\mu_{T_r} = \frac{\ln 2}{2.7 \text{ d}} = 0.25 \text{ d}^{-1}.$$

3.15 Estimate for μ_{I_2}

The half-life of IL-2 ranges between 5–7 minutes [44]. We take $t^{I_2}_{1/2} = 6$ minutes and get

$$\mu_{I_2} = \frac{\ln 2}{4.17 \times 10^{-3} \text{ d}} = 166.22 \text{ d}^{-1}.$$

3.16 Estimate for $\mu_{I_{12}}$

The half-life of IL-12 ranges between 5.3–10.3 hours [45]. We take $t^{I_{12}}_{1/2} = 7.8$ hours. Hence,

$$\mu_{I_{12}} = \frac{\ln 2}{0.325 \text{ d}} = 2.13 \text{ d}^{-1}.$$

3.17 Estimate for μ_{A_4}

The half-life of ipilimumab is 14.7 days [46]. Hence,

$$\mu_{A_4} = \frac{\ln 2}{14.7 \text{ d}} = 4.72 \times 10^{-2} \text{ d}^{-1}.$$

3.18 Estimate μ_S

The half-life of prednisone is 3–4 hours [47], and of methylprednisolone is 2.5–3.5 hours [48]; we take the half-life of prednisone to be 3.5 hours (0.15 days) and for mehtylprednisone to be 3 hours (0.125 days). Hence,

$$\mu_S = \frac{\ln 2}{0.15 \text{ d}} = 4.62 \text{ d}^{-1} \text{ (prednisone), and } \mu_S = \frac{\ln 2}{0.125 \text{ d}} = 5.54 \text{ d}^{-1} \text{ (methylprednisolone).}$$

3.19 Estimates for ε_C

The parameter ε_C increases with the aggressiveness of the cancer; we assume that $\varepsilon_C > 1$ and take

$$\varepsilon_C = 2.$$

3.20 Estimates of production parameters

We estimate production parameters in Eqs. (2)–(10) by assuming steady state equation without treatment.

Eq. (2) We use the steady state equation

$$\lambda_{DC} D_0/2 - \mu_D D = 0,$$

with $\mu_D = 0.13$ d^{-1}, $D_0 = 2 \times 10^{-5}$ g/cm^3 [24], and $D = 4 \times 10^{-4}$ g/cm^3 [24]. Hence,

$$\lambda_{DC} = 5.2 \text{ d}^{-1}.$$

Eqs. (3) **and** (4) We take $\lambda_{T_1 I_{12}} = \lambda_{T_8 I_{12}}$, $\lambda_{T_1 T_\alpha} = 3\lambda_{T_8 T_\alpha}$ and solve simultaneously the following steady states equations without treatment:

$$\left(T_{10} \frac{\lambda_{T_1 I_{12}}/2 + \lambda_{T_1 T_\alpha}/2}{2} + \frac{\lambda_{T_1 I_2}}{2} T_1 \right) /2 - \mu_{T_1} T_1 = 0$$
$$\left(T_{80} \frac{\lambda_{T_8 I_{12}}/2 + \lambda_{T_8 T_\alpha}/2}{2} + \frac{\lambda_{T_8 I_2}}{2} T_8 \right) /2 - \mu_{T_8} T_8 = 0$$

with $\lambda_{T_1 I_2} = \lambda_{T_8 I_2} = 0.25$ d^{-1} [24], $\mu_{T_1} = 0.2$ d^{-1}, $\mu_{T_8} = 0.2$ d^{-1}, $T_{10} = 4 \times 10^{-4}$ g/cm^3, $T_{80} = 2 \times 10^{-4}$ g/cm^3 [24], $T_1 = 2 \times 10^{-3}$ g/cm^3, and $T_8 = 5 \times 10^{-4}$ g/cm^3. Hence,

$$\lambda_{T_1 I_{12}} = \lambda_{T_8 I_{12}} = 1.375 \text{ d}^{-1},\ \lambda_{T_1 T_\alpha} = 4.125 \text{ d}^{-1},\ \lambda_{T_8 T_\alpha} = 1.375 \text{ d}^{-1}.$$

Eq. (5) We use the steady state equation

$$\lambda_{T_r T_\beta} T_{10}/2 - \mu_{T_r} T_r = 0,$$

with $\mu_{T_r} = 0.25$ d^{-1}, $T_{10} = 4 \times 10^{-4}$ g/cm^3 [24], and $T_r = 1.04 \times 10^{-4}$ g/cm^3. Hence,

$$\lambda_{T_r T_\beta} = 0.13 \text{ d}^{-1}.$$

Eq. (6) We use the steady state equation

$$\lambda_C C\,(1 - C/C_M) - \mu_{T_8C} T_8 C - \mu_C C = 0,$$

with $\lambda_C = 0.203$ d^{-1}, $\mu_C = 0.17$ d^{-1}, $C_M = 4.9$ g/cm^3, $C = 0.4$ g/cm^3 [24], and $T_8 = 5 \times 10^{-4}$ g/cm^3. Hence,

$$\mu_{T_8C} = 33 \text{ cm}^3/\text{g}\cdot\text{d}.$$

Eq. (7) We use the steady state equation

$$\lambda_{I_2T_1} T_1 - \mu_{I_2} I_2 = 0,$$

with $\mu_{I_2} = 166.22$ d^{-1}, $I_2 = K_{I_2} = 1.9 \times 10^{-11}$ g/cm^3, and $T_1 = 2 \times 10^{-3}$ g/cm^3. Hence,

$$\lambda_{I_2T_1} = 1.6 \times 10^{-6} \text{ d}^{-1}.$$

Eq. (8) We use the steady state equation

$$\lambda_{I_{12}D} D - \mu_{I_{12}T_1} T_1/2 - \mu_{I_{12}} I_{12} = 0,$$

with $\mu_{I_{12}} = 2.13$ d^{-1}, $\mu_{I_{12}T_1} = 10^{-7}$ d^{-1}, $D = 4 \times 10^{-4}$ g/cm^3, and $I_{12} = K_{I_{12}} = 10^{-10}$ g/cm^3. Hence,

$$\lambda_{I_{12}D} = 3.03 \times 10^{-6} \text{ d}^{-1}.$$

Eq. (9) We use the steady state equation

$$\lambda_{T_\alpha T_1} T_1 - \mu_{T_\alpha} T_\alpha = 0,$$

with $\mu_{T_\alpha} = 199$ d^{-1}, $T_\alpha = K_{T_\alpha} = 8.4 \times 10^{-12}$ g/cm^3, and $T_1 = 2 \times 10^{-4}$ g/cm^3. Hence,

$$\lambda_{T_\alpha T_1} = 8.4 \times 10^{-7} \text{ d}^{-1}.$$

Eq. (10) We take $\lambda_{T_\beta C} = 2\lambda_{T_\beta T_r}$, and solve the following steady state equation

$$\lambda_{T_\beta T_r}(2C + T_r) - \mu_{T_\beta} T_\beta = 0,$$

with $\mu_{T_\beta} = 399.25$ d^{-1}, $T_\beta = K_{T_\beta} = 7.2 \times 10^{-9}$ g/cm^3, $T_r = 1.04 \times 10^{-4}$ g/cm^3 and $C = K_C = 0.4$ g/cm^3 [24]. Hence,

$$\lambda_{T_\beta C} = 7.2 \times 10^{-6} \text{ d}^{-1},\ \lambda_{T_\beta T_r} = 3.6 \times 10^{-6} \text{ d}^{-1}.$$

4 Parameter Sensitivity Analysis

We performed sensitivity analysis with respect to the tumor volume, for a group of parameters which were roughly estimated. We first established the important parameters by performing a global sensitivity analysis with most parameters, especially those that represent activation, transition or absorption rates, any by retaining those with significant PRCC and p-value less than 0.05 (see Figure 1). We then performed sensitivity analysis with these selected parameters (see Figure 2).

The computations were done using Latin Hypercube Sampling/Partial Rank Correlation Coefficient (LHS/PRCC) with a Matlab package by [49, 50]. The range for the parameters in the sensitivity analysis were between ±50% of their baseline values in

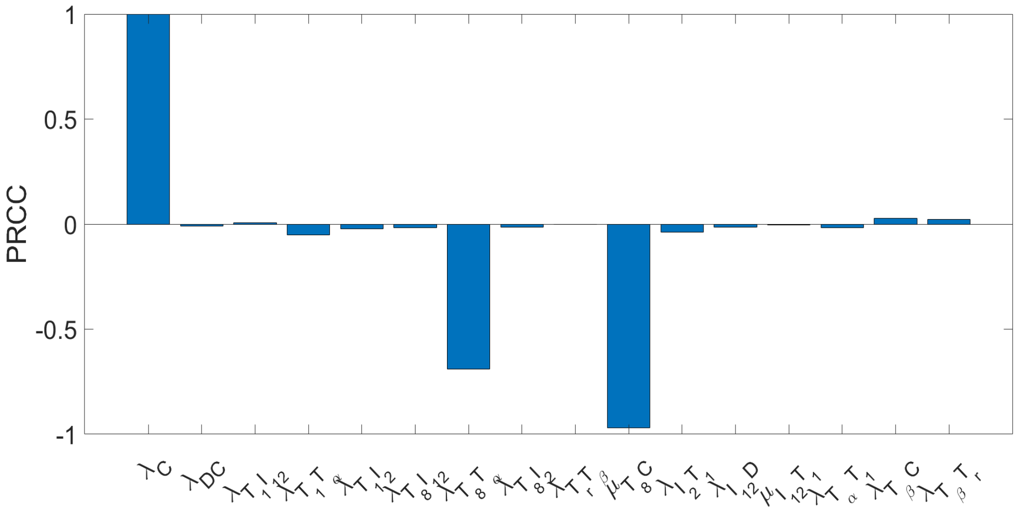

Fig 1. Parameter sensitivity analysis for the tumor volume at day 70 with all the activation, transition and absorption parameters. We retain the parameters λ_C. $\lambda_{T_8T_\alpha}$ and μ_{T_8C} whose p-values are less than 0.01. All other parameters have p-values > 0.1 and are discarded.

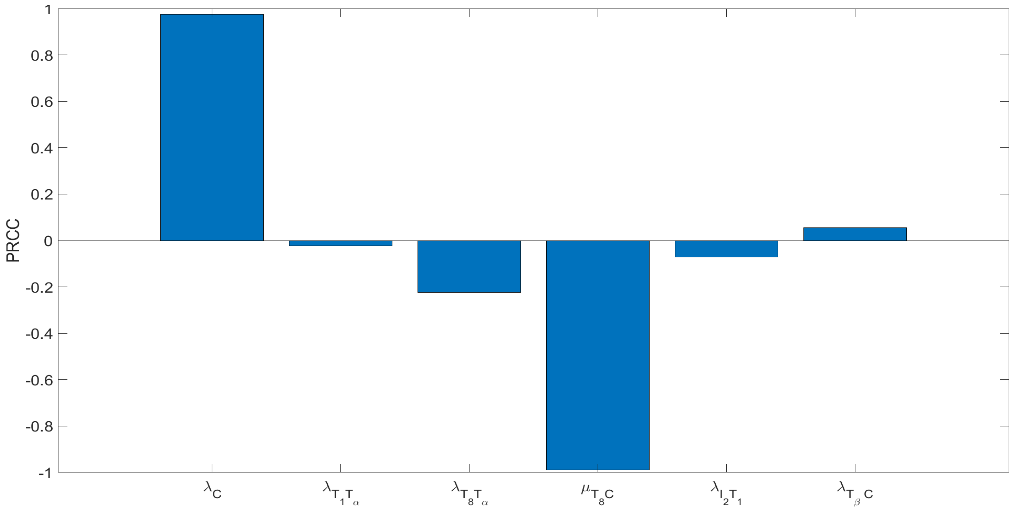

Fig 2. Parameter sensitivity analysis for the tumor volume at day 70. The p-value is less than 0.01.

Tables 1–2, except for λ_C and μ_{T_8C} which were chosen between $\pm5\%$ of the baseline in Table 1.

The proliferation rate of C, λ_C, is clearly positively correlated, while the killing rate of C by T_8 cells, μ_{T_8C} is clearly negatively correlated; so are $\lambda_{T_8T_\alpha}$, $\lambda_{T_1T_\alpha}$ and $\lambda_{I_2T_1}$, since an increase in either T_1 or T_8 is anti-cancer. The parameter $\lambda_{T_\beta C}$ is positively correlated since an increase in this parameter increases the proliferation of T_r which controls T_1 and T_8.

5 Numerical Method

We used the moving mesh method [51] to numerically solve the free boundary problem for the tumor proliferation model. To illustrate in this model, we take Eq. (6) as example and rewrite it in the following form:

$$\frac{\partial C(r,t)}{\partial t} = \delta_C \Delta C(r,t) - \nabla \cdot (\mathbf{u}C) + F, \tag{23}$$

where F represents the term in the right-hand side of Eq. (6). Let r_i^k and C_i^k denote numerical approximations of i-th grid point and $C(r_i^k, n\tau)$, respectively, where τ is the size of time-step. The discretization of Eq. (23) is derived by the fully implicit finite difference scheme:

$$\frac{C_i^{k+1} - C_i^k}{\tau} = \delta_C \left(C_{rr} + \frac{2}{r_i^k} C_r \right) - \left(\frac{2}{r_i^{k+1} u_i^{k+1} + u_r} \right) C_i^{k+1} - u_i^{k+1} C_r + F_i^{k+1},$$

where $C_r = \frac{h_{-1}^2 C_{i+1}^{k+1} - h_1^2 C_{i-1}^{k+1} - (h_1^2 - h_{-1}^2) C_i^{k+1}}{h_1 (h_{-1}^2 - h_1 h_{-1})}$, $C_{rr} = 2\frac{h_{-1} C_{i+1}^{k+1} - h_1 C_{i-1}^{k+1} + (h_1 - h_{-1}) C_1^{k+1}}{h_1 (h_1 h_{-1} - h_{-1}^2)}$,
$u_r = \frac{h_{-1}^2 u_{i+1}^{k+1} - h_1^2 u_{i+1}^{k+1} - (h_1^2 - h_{-1}^2) u_i^{k+1}}{h_1 (h_{-1}^2 - h_1 h_{-1})}$, $h_{-1} = r_{i-1}^{k+1} - r_i^{k+1}$ and $h_1 = r_{i+1}^{k+1} - r_i^{k+1}$. The mesh moves by $r_i^{k+1} = r_i^k + u_i^{k+1}\tau$, where u_i^{k+1} is solved by the velocity equation.

In order to make the scheme stable, we take $\tau \leq \frac{h^2}{4\delta_C}$, namely $\tau = 0.1\frac{h^2}{\delta_C}$, where $h = \min(h_{-1}, h_1)$.

In the case of cytokines, the advection term is negligible compared to the large diffusion of proteins, and is dropped.

Table 1. Parameters for the model.

Parameters	Descriptions	Values	references
λ_C	proliferation rate of C	0.203 d^{-1}	[52]est.
C_M	carrying capacity for N and M	4.9 g/cm^3	fitted
D_0	source of D	2×10^{-5} g/cm^3	[24]
T_{10}	source of T_1	4×10^{-4} g/cm^3	[24]
T_{80}	source of T_8	2×10^{-4} g/cm^3	[24]
$\hat{T}_1$	inflow of T_1 from lymph node	4×10^{-3} g/cm^3	[24]
$\hat{T}_8$	inflow of T_8 from lymph node	2×10^{-3} g/cm^3	[24]
μ_C	rate of death of C	0.17 d^{-1}	[53]est.
μ_D	rate of death of D	0.13 d^{-1}	[41]est.
μ_{T_1}	rate of death of T_1	0.2 d^{-1}	[42]est.
μ_{T_8}	rate of death of T_8	0.2 d^{-1}	[42]est.
μ_{T_r}	rate of death of T_r	0.25 d^{-1}	[43]est.
μ_{I_2}	rate of decay of I_2	166.22 d^{-1}	[44]est.
$\mu_{I_{12}}$	rate of decay of I_{12}	2.13 d^{-1}	[45]est.
μ_{T_α}	rate of decay of T_α	199 d^{-1}	[54, 55] est.
μ_{T_β}	rate of decay of T_β	399.25 d^{-1}	[56]
μ_{A_4}	decay rate of A_4	4.72×10^{-2} d^{-1}	[46]est.
μ_S	decay rate of S	4.62/5.54 d^{-1}	[47, 48]est.
μ_Q	decay rate of Q	6×10^4 d^{-1}	[20]
$\delta_C,\ \delta_D,\ \delta_T$	diffusion coefficient of cells	8.64×10^{-7} $cm^2 d^{-1}$	[24]est.
δ_{I_2}	diffusion coefficient of I_2	9.92×10^{-2} $cm^2 d^{-1}$	[34, 35]est.
$\delta_{I_{12}}$	diffusion coefficient of I_{12}	7.5×10^{-2} $cm^2 d^{-1}$	[34, 35]est.
δ_{T_α}	diffusion coefficient of T_α	9.76×10^{-2} $cm^2 d^{-1}$	[35, 57] est.
δ_{T_β}	diffusion coefficient of T_β	14.86×10^{-2} $cm^2 d^{-1}$	[35, 38]est.
δ_{A_4}	diffusion coefficient of A_4	7.5×10^{-2} $cm^2 d^{-1}$	[35, 39]est.
δ_S	diffusion coefficient of S	3.51×10^{-2} $cm^2 d^{-1}$	[35, 40] est.
$\mu_{T_8 C}$	killing rate of C by T_8	33 $cm^3/g{\cdot}d$	est.
$\mu_{P_A A_4}$	rate of depletion of A_4 by P_A	1.1×10^7 $cm^3/g{\cdot}d$	fitted
μ_{ST}	inhibition rate of T_1 and T_8 by S	5.31×10^6 $cm^3/g{\cdot}d$	fitted
μ_{TS}	absorption rate of S by T_1 and T_8	9×10^3 $cm^3/g{\cdot}d$	fitted
$\mu_{I_{12} T_1}$	decay of I_{12} due to T_1 and T_8	10^{-7} $cm^3/g{\cdot}d$	fitted

est.= this parameter was estimated in Section 3.

Table 2. Parameters for the model (continued).

Parameters	Descriptions	Values	references
λ_{DC}	activation rate of D by C	5.2 d^{-1}	est.
$\lambda_{T_1 I_2}$	proliferation rate of T_1 by I_2	0.25 d^{-1}	[24]
$\lambda_{T_8 I_2}$	proliferation rate of T_8 by I_2	0.25 d^{-1}	[24]
$\lambda_{T_1 I_{12}}$	activation rate of T_1 by I_{12}	1.375 d^{-1}	est.
$\lambda_{T_8 I_{12}}$	activation rate of T_8 by I_{12}	1.375 d^{-1}	est.
$\lambda_{T_1 T_\alpha}$	activation rate of T_1 by T_α	4.125 d^{-1}	est.
$\lambda_{T_8 T_\alpha}$	activation rate of T_8 by T_α	1.375 d^{-1}	est.
$\lambda_{T_r T_\beta}$	activation rate of T_r by T_β	0.13 d^{-1}	est.
$\lambda_{I_2 T_1}$	production rate of I_2 by T_1	1.6×10^{-6} d^{-1}	est.
$\lambda_{I_{12} D}$	production rate of I_{12} by D	3.03×10^{-6} d^{-1}	est.
$\lambda_{I_{12} A_4}$	production rate of I_{12} due to A_4	10^{-3} d^{-1}	est.
$\lambda_{T_\alpha T_1}$	production rate of T_α by T_1	8.4×10^{-7} d^{-1}	fitted
$\lambda_{T_\beta C}$	production rate of T_β by C	7.2×10^{-6} d^{-1}	est.
$\lambda_{T_\beta T_r}$	production rate of T_β by T_r	3.6×10^{-6} d^{-1}	est.
K_D	half saturation of D	4×10^{-4} g/cm^3	[24]
K_{TT_r}	inhibition of T_1 and T_8 by T_r	1.04×10^{-4} g/cm^3	[23]est.
K_{I_2}	half saturation of I_2	1.9×10^{-11} g/cm^3	[58]est.
$K_{I_{12}}$	half saturation of I_{12}	10^{-10} g/cm^3	[58]est.
K_{T_α}	half saturation of T_α	8.4×10^{-12} g/cm^3	[57]est.
K_{T_β}	half saturation of T_β	7.2×10^{-9} g/cm^3	[56]
K_Q	half saturation of Q	4.86×10^{-20} g^2/cm^6	[24]
K'_{TQ}	inhibition of T_1 and T_8 by P_A-B_7	4.86×10^{-20} g^2/cm^6	[24]
θ	constant density of cells	0.5 g/cm^3	est.
κ_T	(#P_A per T_r)/(#P_A per T_1 or T_8)	1	est.

est.= this parameter was estimated in Section 3.

References

1. Sims GP, Rowe DC, Rietdijk ST, Herbst R, Coyle AJ. Hmgb1 and rage in inflammation and cancer. Annu Rev Immunol. 2010;28:367–388.

2. Palucka J, Banchereau J. Cancer immunotherapy via dendritic cells. Nat Rev Cancer. 2012;12(4):265–277.

3. Saenz R, Futalan D, Leutenez L, Eekhout F, Fecteau JF, Sundelius S, et al. Tlr4-dependent activation of dendritic cells by an hmgb1-derived peptide adjuvant. J Transl Med. 2014;12(211):1–11.

4. Yamane H, Igarashi O, Kato T, Nariuchi H. Positive and negative regulation of IL-12 receptor expression of naive CD4+T cells by CD28/CD152 co-stimulation. Eur J Immunol. 2000;30:3171–3180.

5. Mehta AK, Gracias DT, Croft M. TNF activity and T cells. Cytokine. 2018;101:14–18. doi:10.1016/j.cyto.2016.08.003.

6. Whiteside TL. The role of regulatory t cells in cancer immunology. Immunotargets Ther. 2015;4:159–171.

7. Nelson BH. IL-2, regulatory T cells, and tolerance. J Immunol. 2004;172(7):3983–3988.

8. Ross SH, Cantrell DA. Signaling and Function of Interleukin-2 in T Lymphocytes. Annu Rev Immunol. 2018;36:411–433. doi:10.1146/annurev-immunol-042617-053352.

9. Schuyler MR, Gerblich A, Urda G. Prednisone and T-Cell Subpopulations. Arch Intern Med. 1984;144(5):973–975. doi:10.1001/archinte.1984.00350170119021.

10. McNally A, Hill GR, Sparwasser T, Thomas R, Steptoe RJ. CD4+CD25+ regulatory T cells control CD8+ T-cell effector differentiation by modulating IL-2 homeostasis. Proc Natl Acad Sci U S A. 2011;108(18):7529–34. doi:10.1073/pnas.1103782108.

11. Tran DQ. TGF-β: the sword, the wand, and the shield of FOXP3(+) regulatory T cells. J Mol Cell Biol. 2012;4(1):29–37. doi:10.1093/jmcb/mjr033.

12. Ma Y, Shurin GV, Peiyuan Z, Shurin MR. Dendritic cells in the cancer microenvironment. J Cancer. 2013;4(1):36–44.

13. Janco JMT, Lamichhane P, Karyampudi L, Knutson KL. Tumor-infiltrating dendritic cells in cancer pathogenesis. J Immunol. 2015;194(7):2985–2991.

14. Krishnan T, Tomita Y, Roberts-Thomson R. A retrospective analysis of eosinophilia as a predictive marker of response and toxicity to cancer immunotherapy. Future Sci OA. 2020;6(10):FSO608. doi:10.2144/fsoa-2020-0070.

15. RELX Group (TM). Interleukin 12: Interleukin-12 (IL-12) is a potent proinflammatory cytokine that enhances the cytotoxic activity of T lymphocytes and resting natural killer cells. Pediatric Surgery, Seventh Ed. 2012;217.

16. Perrot CY, Javelaud D, Mauviel A. Insights into the transforming growth factor-beta signaling pathway in cutaneous melanoma. Ann Dermatol. 2013;25(2):135–144.

17. Ha D, Tanaka A, Kibayashi T, Tanemura A, Sugiyama D, Wing JB, et al. Differential control of human Treg and effector T cells in tumor immunity by Fc-engineered anti–CTLA-4 antibody. PNAS. 2019;116(2):609–618.

18. Du X, Tang F, Liu M, Su J, Zhang Y, Wu W, et al. A reappraisal of CTLA-4 checkpoint blockade in cancer immunotherapy. Cell Res. 2018;28:416–432. doi:10.1038/s41422-018-0011-0.

19. Rudd CE. CTLA-4 co-receptor impacts on the function of Treg and CD8+ T-cell subsets. Eur J Immunol. 2012;39(3):687–690. doi:10.1002/eji.200939261.

20. Maute RL, Gordon SR, Mayer AT, McCracken MN, Natarajan A, Ring NG, et al. Engineering high-affinity PD-1 variants for optimized immunotherapy and immuno-PET imaging. Proc Natl Acad Sci USA. 2015;112(47):E6506–14.

21. Cheng X, Veverka V, Radhakrishnan A, Waters LC, Muskett FW, Morgan SH, et al. Structure and interactions of the human programmed cell death 1 receptor. J Biol Chem. 2013;288(17):11771–11785.

22. Lisiero DN, Soto H, Liau LM, Prins RM. Enhanced sensitivity of IL-2 signaling regulates the clinical responsiveness of IL-12-primed $CD8^+$ T cells in a melanoma model. J Immunol. 2011;186:5068–5077.

23. Eckert F, Schaedle P, Zips D, Schmid-Horche B, Rammensee H, Gani C, et al. Impact of curative radiotherapy on the immune status of patients with localized prostate cancer. Oncoimmunol. 2018;7(11):e1496881 (11 pages). doi:10.1080/2162402X.2018.1496881.

24. Lai X, Stiff A, Duggan M, Wesolowski R, Carson III WE, Friedman A. Modeling combination therapy for breast cancer with BET and immune checkpoint inhibitors. PNAS. 2018;115(21):5534–5539.

25. Kucera R, Topolcan O, Treskova I, Kinkorova J, Windrichova J, Fuchsova R, et al. Evaluation of IL-2, IL-6, IL-8 and IL-10 in Malignant Melanoma Diagnostics. Anticancer Res. 2015;35(6):3537–3541.

26. Jafarzadeh A, Minaee K, Farsinejad A, Nemati M, Khosravimashizi A, Daneshvar H, et al. Evaluation of the circulating levels of IL-12 and IL-33 in patients with breast cancer: influences of the tumor stages and cytokine gene polymorphisms. Iran J Basic Med Sci. 2015;18(12):1189–1198.

27. Imani MM, Sadeghi M, Khazaie H, Emami M, Bahmani DS, Brand S. Serum and Plasma Tumor Necrosis Factor Alpha Levels in Individuals with Obstructive Sleep Apnea Syndrome: A Meta-Analysis and Meta-Regression. Life (Basel). 2020;10(6):87. doi:10.3390/life10060087.

28. Kim HO, Kim H, Youn J, Shin E, Park S. Serum cytokine profiles in healthy young and elderly population assessed using multiplexed bead-based immunoassays. J Transl Med. 2011;9(113). doi:10.1186/1479-5876-9-113.

29. Li G, Wu W, Zhang X, Huang Y, Wen Y, Li X, et al. Serum levels of tumor necrosis factor alpha in patients with IgA nephropathy are closely associated with disease severity. BMC Nephrol. 2018;19(326). doi:10.1186/s12882-018-1069-0.

30. Marques-Vidal P, Bochud M, Bastardot F, Lüscher T, Ferrero F, Gaspoz JM, et al. Levels and determinants of inflammatory biomarkers in a Swiss population-based sample (CoLaus study). PLoS One. 2011;6(6):e21002. doi:10.1371/journal.pone.0021002.

31. Song XY, Zhou SJ, Xiao N, Li YS, Zhen DZ, Su CY, et al. Research on the relationship between serum levels of inflammatory cytokines and non-small cell lung cancer. Asian Pac J Cancer Prev. 2013;14(8):4765–4768. doi:10.7314/apjcp.2013.14.8.4765.

32. Berking C, Takemoto R, Schaider H, Showe L, Satyamoorthy K, Robbins P, et al. Transforming Growth Factor-β1 Increases Survival of Human Melanoma through Stroma Remodeling. Cancer Res. 2001;61:8306–8316.

33. Krombach F, Münzing S, Allmeling AM, Gerlach JT, Behr J, M MD. Cell size of alveolar macrophages: an interspecies comparison. Environ Health Perspect. 1997;105(5):1261–1265. doi:10.1289/ehp.97105s51261.

34. Young ME. Estimation of diffusion coefficients of proteins. Biotech Bioeng. 1980;XXII:947–955.

35. Hornbeck PV, Zhang B, Murray B, Kornhauser JM, Latham V, Skrzypek E. PhosphoSitePlus, 2014: mutations, PTMs and recalibrations. Nucleic Acids Research. 2015;43:D512–D520.

36. Liao KL, Bai XF, Friedman A. Mathematical modeling of interleukin-27 induction of anti-tumor T cells response. PLoS ONE. 2014;9(3).

37. GenScript. TNF-α, Human. https://wwwgenscriptcom/protein/Z01001-TNF_Humanhtml. Accessed September 29, 2021;.

38. National Center for Biotechnology Information. PubChem Compound Summary for CID 56842206, TGF-beta. PubChem, https://pubchemncbinlmnihgov/compound/TGF-beta. Accessed January 24, 2021;.

39. Abcam. Recombinant Anti-CTLA4 antibody [EPR1476] (ab134090). https://wwwabcamcom/ctla4-antibody-epr1476-ab134090html;.

40. National Center for Biotechnology Information. PubChem Compound Summary for CID 5755, Prednisolone. https://pubchemncbinlmnihgov/compound/Prednisolone. Accessed September 29, 2021;.

41. Diao J, Winter E, Cantin C, Chen W, Xu L, Kelvin D, et al. In Situ Replication of Immediate Dendritic Cell (DC) Precursors Contributes to Conventional DC Homeostasis in Lymphoid Tissue. J Immunol. 2006;176(12):7196–7206. doi:10.4049/jimmunol.176.12.7196.

42. Rocha B, Freitas AA, Coutinho AA. Population dynamics of T lymphocytes. Renewal rate and expansion in the peripheral lymphoid organs. J Immunol. 1983;131(5):2158–2164.

43. Furlan SN, Singh K, Lopez C, Tkachev V, Hunt DJ, Hibbard J, et al. IL-2 enhances ex vivo–expanded regulatory T-cell persistence after adoptive transfer. Blood Adv. 2020;4(8):1594–1605.

44. Lotze MT, Frana LW, Sharrow SO, Robb RJ, Rosenberg SA. In vivo administration of purified human interleukin 2. I. Half-life and immunologic effects of the Jurkat cell line-derived interleukin 2. J Immunol. 1985;134(1):157–166.

45. Jung K, Ha J, Kim J, Kim J, Kim Y, Kim C, et al. Heterodimeric Fc-fused IL12 shows potent antitumor activity by generating memory CD8+ T cells. OncoImmunol. 2018;7(7).

46. Fellner C. Ipilimumab (yervoy) prolongs survival in advanced melanoma: serious side effects and a hefty price tag may limit its use. P & T. 2012;37(9):503–530.

47. Drugs com. How long does it take prednisone to get out of your system? https://wwwdrugscom/medical-answers/prednisone-how-long-does-it-take-predesone-to-get-372449/. Last updated on March 22, 2021;.

48. Drugs com. Medrol Dosepak - How long does a dose pack stay in your system? 2020;.

49. Kirschner DE. Uncertainty and sensitivity functions and implementation. http://malthus.micro.med.umich.edu/lab/usadata/: University of Michigan; 2007–2008.

50. Marino S, Hogue IB, Ray CJ, Kirschner DE. A methodology for performing global uncertainty and sensitivity analysis in systems biology. J Theor Biol. 2008;254:178–196.

51. D'Acunto B. Computational Methods for PDE in Mechanics, Series on Advances in Mathematics for Applied Sciences. World Scientific. 2004;67.

52. Linxweiler J, Körbel C, Müller A, Hammer M, Veith C, Bohle RM, et al. A novel mouse model of human prostate cancer to study intraprostatic tumor growth and the development of lymph node metastases. Prostate. 2018;78(9):664–675. doi:10.1002/pros.23508.

53. Lemech C, Arkenau HT. Novel treatments for metastatic cutaneous melanoma and the management of emergent toxicities. Clin Med Insights Oncol. 2012;6:53–66. doi:10.4137/CMO.S5855.

54. Friedman A, Siewe N. Chronic Hepatitis B Virus and Liver Fibrosis: A Mathematical Model. PLoS ONE. 2018;13(4):1–23.

55. Simo R, Barbosa-Desongles A, Lecube A, Hernandez C, Selva DM. Potential Role of Tumor Necrosis Factor-α in Downregulating Sex Hormone–Binding Globulin. Diabetes. 2012;61:372–382.

56. Siewe N, Friedman A. TGF-β inhibition can overcome cancer primary resistance to PD-1 blockade: a mathematical model. PLoS ONE. 2021;16(6):1–16.

57. Michalaki V, Syrigos K, Charles P, Waxman J. Serum levels of IL-6 and TNF-α correlate with clinicopathological features and patient survival in patients with prostate cancer. Br J Cancer. 2004;90:2312–2316. doi:10.1038/sj.bjc.6601814.

58. Tazaki E, Shimizu N, Tanaka R, Toshizumi M, Kamma H, Imoto S, et al. Serum cytokine profiles in patients with prostate carcinoma. Exp Ther Med. 2011;2(5):887–891. doi:10.3892/etm.2011.286.
