# Supplementary material for: Optimal timing of steroid initiation in response to CTLA-4 antibody in metastatic cancer: A mathematical model
Source: PLoS One. 2022 Nov 10;17(11):e0277248. doi: 10.1371/journal.pone.0277248 (PMC9648769; doi:10.1371/journal.pone.0277248)
Supplement: S1 Appendix — (PDF) [file pone.0277248.s002.pdf]

Appendix

Copy of Figs. 1A,B and 3H in our reference [70]

The following figure is taken from Figures 1A,B and 3H in: *Tokunaga A, Sugiyama D, Maeda Y, Warner AB, Panageas KS, Ito S, et al. Selective inhibition of low-affinity memory CD8^{+} T cells by corticosteroids. J Exp Med. 2019;216(12):2701–2713. doi:10.1084/jem.20190738.*

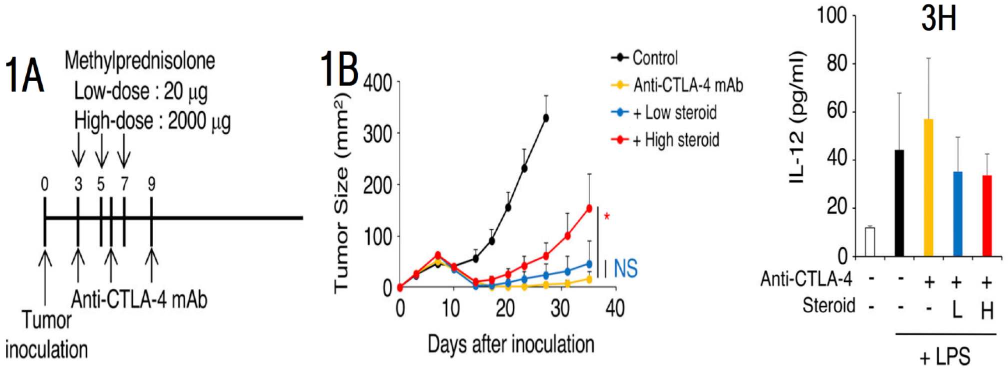

Fig 12. Copy of Figs. 1A,B and 3H in our reference [70].

Copy of Figs. 6a,b in our reference [9]

The following figures are taken from Figure 6 in: *Giles AJ, Hutchinson MND, Sonnemann HM, Jung J, Fecci PE, Ratman NM, et al. Dexamethasone-induced immunosuppression: mechanisms and implications for immunotherapy. J Immunother Cancer. 2018;6(1):51. doi:10.1186/s40425-018-0371-5.*

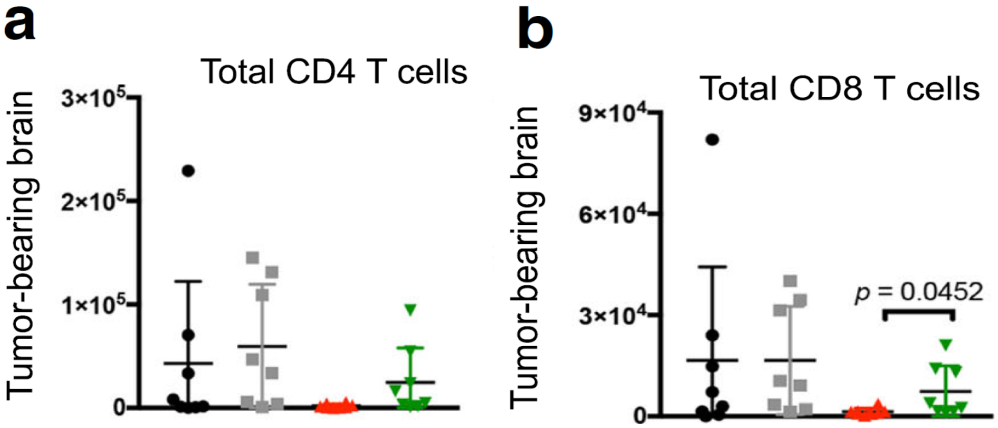

Fig 13. Copy of Figs. 6a,b in in our reference [9].
